# Comprehensive study of nuclear receptor DNA binding provides a revised framework for understanding receptor specificity

**DOI:** 10.1038/s41467-019-10264-3

**Published:** 2019-06-07

**Authors:** Ashley Penvose, Jessica L. Keenan, David Bray, Vijendra Ramlall, Trevor Siggers

**Affiliations:** 1 Department of Biology, Boston University, Boston, MA 02215 USA; 2 Biological Design Center, Boston University, Boston, MA 02215 USA; 3 Bioinformatics Program, Boston University, Boston, MA 02215 USA

**Keywords:** DNA, Proteins, Transcriptional regulatory elements, Systems biology

## Abstract

The type II nuclear receptors (NRs) function as heterodimeric transcription factors with the retinoid X receptor (RXR) to regulate diverse biological processes in response to endogenous ligands and therapeutic drugs. DNA-binding specificity has been proposed as a primary mechanism for NR gene regulatory specificity. Here we use protein-binding microarrays (PBMs) to comprehensively analyze the DNA binding of 12 NR:RXRα dimers. We find more promiscuous NR-DNA binding than has been reported, challenging the view that NR binding specificity is defined by half-site spacing. We show that NRs bind DNA using two distinct modes, explaining widespread NR binding to half-sites in vivo. Finally, we show that the current models of NR specificity better reflect binding-site activity rather than binding-site affinity. Our rich dataset and revised NR binding models provide a framework for understanding NR regulatory specificity and will facilitate more accurate analyses of genomic datasets.

## Abstract (web summary)

The type II nuclear receptors (NRs) and the retinoid X receptor (RXR) form heterodimeric transcription factors to regulate development, metabolism, and inflammation. Here the authors employ protein-binding microarrays to comprehensively analyze the DNA binding of 12 NR:RXRα heterodimers, and report promiscuous NR-DNA binding.

## Introduction

The type II nuclear receptors (hereafter simply NRs) are ligand-activated transcription factors (TFs) that control diverse cellular processes including development, metabolism, and inflammation^1,2^. NRs include peroxisome-proliferator activated receptor (PPAR), liver x receptor (LXR), retinoic acid receptor (RAR), farnesoid x receptor (FXR), pregnane x receptor (PXR), thyroid hormone receptor (THR), and vitamin D receptor (VDR)^2,3^. NRs function as heterodimers with the common partner, the retinoid x receptor (RXR). Individual NR heterodimers can regulate distinct gene programs^4,5^; however, the current models of NR-DNA binding specificity are insufficient to explain NR-specific gene regulation.

**Fig. 1:**
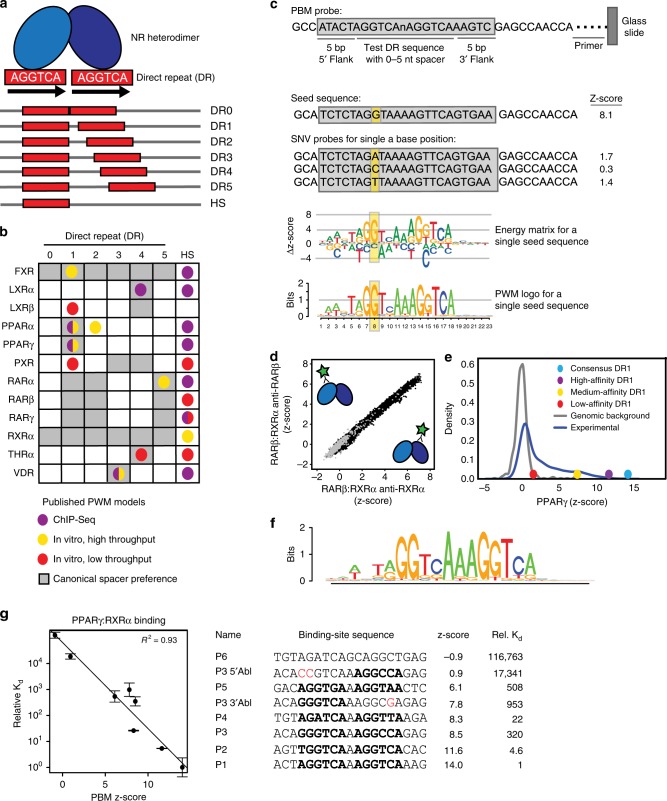
Characterizing NR-DNA binding with PBMs. **a** Schematic of spacer preferences for NRs to direct repeats (DRs) and half-sites (HS). **b** Canonical spacer preferences of NRs indicate preferred spacer lengths from the literature (Supplementary Data 2 and 3). Published PWM models are shown in colored dots that indicate the methodology used to derive the model (Supplementary Data 3). **c** Schematic of PBM probes, SNV probe organization and SNV-based motif generation for a single seed sequence. **d** Scatter plot of z-scores for RARβ:RXRα experiments detected with antibodies against each heterodimer partner. Dots represent average over ~5 replicates for all 10,728 unique SNV probes (black dots) and 500 background probes (gray dots) **e** PBM replicate averaged z-score distributions for PPARγ:RXRα to all SNV probes. Z-scores for consensus DR1 and reported functional binding sites are highlighted (Supplementary Data 2)^6^. **f** DR1 DNA-binding logo for PPARγ:RXRα generated from all DR1 full-site models from the PBM experiments. **g** Comparison of PPARγ:RXRα PBM z-scores and competition EMSA-determined relative K_d_ measurements for binding sites spanning a wide affinity range. Relative K_d_ values are normalized to the highest affinity sequence (P1) and represent mean over two independent experiments (error bars = STDEV). Identifiable DR half-sites in each binding sequence are shown in bold. Mutations introduced to ablate the 5’ half-site of P3 (P3 5’Abl) or the 3’ half-site of P3 (P3 3’Abl) are shown in red. Source data are provided as a Source Data file

NRs bind the sequence 5’-RGKTCA-3’ organized as direct repeats with a variable length spacer of 0–5 base pairs (bp) (DR0-DR5, Fig. 1a)^7–9^. Current models propose that DR spacer length is a key determinant of DNA-binding specificity for NRs^2,8,10–12^. For example, PPAR:RXR dimers prefer binding to DR1 elements, whereas LXR:RXR dimers prefer DR4 elements (Fig. 1b). However, there are more NRs than available spacer lengths; therefore, either DRs are bound by multiple NRs, which presents a problem for achieving NR-specific gene activation, or there are additional determinants of NR-binding specificity beyond DR spacer length.

Differences in DNA-binding specificity for each NR would provide a mechanism for NRs to regulate distinct target genes in vivo. Genome-wide chromatin immunoprecipitation followed by sequencing (ChIP-seq) studies have confirmed known NR preferences for particular DR spacer lengths, and have reinforced the connection between in vitro and in vivo binding^13–20^. However, these studies have also revealed limitations to current models of NR-DNA binding. For example, PPARγ and LXRα regulate distinct yet overlapping gene programs but do not share a DR element to explain their many common genomic targets^13,16^. Additionally, many genomic regions that are bound in vivo lack an identifiable binding site for the NR being investigated (e.g., 90–96% for PPARγ and LXR)^13^. Together, these observations suggest that current models of NR-DNA-binding specificity are incomplete.

To address the need for revised models of NR binding, we use protein-binding microarrays (PBMs) to compare the binding of 12 NR:RXRα dimers to thousands of DNA sequences. To examine DR spacer preferences, we assay NR binding at all spacer lengths (DR0-DR5). We identify both NR-shared and NR-specific binding features in our dataset, and discuss implications for NR-signaling specificity. By integrating PBM and ChIP-seq datasets, we examine the relationship between in vitro and in vivo binding. We address the role of activity versus affinity in current models of NR specificity by integrating PBM data with reporter gene experiments. Our results demonstrate the limitations of DR spacer length for defining NR specificity and of DNA binding affinity for predicting functional binding events.

## Results

### Characterizing NR heterodimer binding with PBMs

We used PBMs to characterize the DNA binding of 12 distinct RXR heterodimers (hereafter NRs). PBMs are double-stranded DNA microarrays that enable the high-throughput study of protein-DNA binding^21^. To characterize both DNA-base and DR-spacing preferences, we measured NR binding to over 1600 unique sequences at each of six DR spacer lengths (DR0-DR5). For each DR spacer length, we measured NR binding to 24 starting sequences, which we refer to as seed sequences (Fig.1c). Seed sequences were generated by combining different half-site sequences exhibiting a range of degeneracy from the consensus 5’-RGKTCA-3’. Most seed sequences contain two distinct half-site sequences. To assay NR binding specificity for each seed sequence we also measured binding to all possible single-nucleotide variants (SNVs), with each SNV included as a separate probe on the PBM (Fig. 1c). This SNV-based approach allows us to generate a binding logo (i.e., energy matrix or position-weight matrix (PWM)) for each individual seed sequence by measuring the impact on binding caused by perturbation at each base position (Fig. 1c, Methods). To capture binding preferences for DR spacer and flank sequences, we included SNVs across the spacer sequence and for the five nucleotides upstream and downstream of the DR. Using this comprehensive SNV-type PBM design, we characterized the DNA-base and DR-spacing preferences of the NRs.

PBM experiments for NR heterodimers (NR:RXRα) were performed by combining purified RXRα with purified samples of each partner NR. Hereafter, we refer to NR:RXRα heterodimers simply by the NR partner, and RXRα:RXRα homodimers as RXRα, unless otherwise stated. Most NRs do not bind DNA with high affinity as homodimers; therefore, proteins were combined at a 3:1 NR:RXRα ratio to force RXRα heterodimer formation (exceptions indicated in Supplementary Data 1). To ensure heterodimer binding, we required that the binding results agreed when performed using antibodies for both RXRα and the non-RXRα partner. Binding profiles using separate antibodies showed strong correlation, demonstrating that both protein partners were bound to each DNA probe at similar levels (Fig. 1d, R^2^ of antibody replicates in Supplementary Data 1). Binding of homodimers were not correlated with each other, nor with the heterodimers (Supplementary Fig. 1), further demonstrating heterodimer binding. To quantify binding specificity, PBM fluorescence values were converted into z-scores using a set of 500 random genomic background sequences (Fig. 1e). Validated PPARγ binding sites score significantly above background, down to a z-score of 1.5 (Fig. 1e). We set a more stringent z-score cutoff of 3.0 to define the affinity cutoff for functional binding sites. A DR1 DNA binding logo generated for PPARγ agrees well with known logos from ChIP-seq (Fig. 1f), demonstrating the sensitivity of our assay. To validate our PBM results with an orthogonal approach, we used competition electrophoretic-mobility shift assays (EMSAs) to measure the relative binding affinity of PPARγ:RXRα to DNA sequences bound over a wide range of PBM z-scores (Fig. 1g, Supplementary Fig. 2). We find strong agreement between the relative binding affinities derived using both approaches (R^2^ = 0.93). Our protein samples were produced in bacterial or insect cells; however, our ability to capture known NR-binding specificity suggests our data reflect native mammalian dimer-binding specificity. These results demonstrate that our PBMs accurately capture sequence-specific binding of NR heterodimers.

### NRs bind promiscuously to most DR spacings

To understand NR-signaling specificity, studies have examined the DNA-binding differences between NRs (summarized in Fig. 1b, Supplementary Data 2 and 3)^2,6,8,11,22^. A prevailing view is that NRs are distinguished by their preference for DR sites with specific half-site spacing^2,8,11,12,22–24^; however, individual NRs are functional on DR sites with various spacings^25–27^. Therefore, for each NR we examined which DR spacings were bound with sufficient affinity such that they might be functional in vivo.

**Fig. 2:**
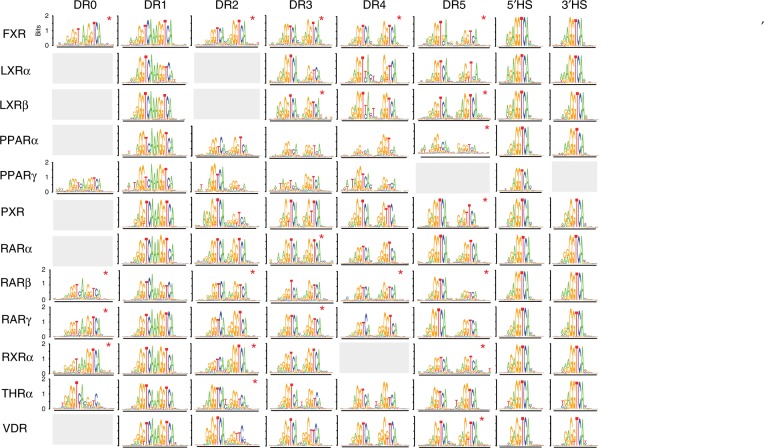
NR-binding specificity and DR preferences. PBM-derived DNA-binding logos for 12 NRs at all examined DR spacer lengths. Half-site logos identified for each NR on either the 5’ half-site (5’HS) or 3’ half-site (3’HS) are shown. Logos based upon a single significant (z-score > 3.0) seed sequence are indicated (∗). Source data are provided as a Source Data file

To visualize the NR-binding landscape, we generated a DNA-binding logo from high-scoring seeds at each DR spacing (Fig. 2). Strikingly, for all NRs we were able generate DNA-binding logos at nearly every DR spacing, demonstrating broader binding preferences than previously reported. Comparing our logos with published DR binding preferences (Fig. 1b), we find high-affinity binding for many NRs at new DR spacings. The binding logos for all NRs exhibit the canonical 5’-RGKTCA-3’ sequence preferences in each half-site and agree with base preferences reported by other methods^28–30^. The logo similarity demonstrates broad conservation in NR-binding specificity; however, NR-specific preferences are also present. For example, PPARγ prefers an AT-rich sequence 5’ of the first half-site of a DR^6^ and our PPARγ logo shows this extended footprint (Figs. 1f, 2). Overall, our data reveal that all NR heterodimers can bind to sites with variable DR spacings and with highly overlapping base specificities.

### All type II NRs can bind DNA using a half-site mode

**Fig. 3:**
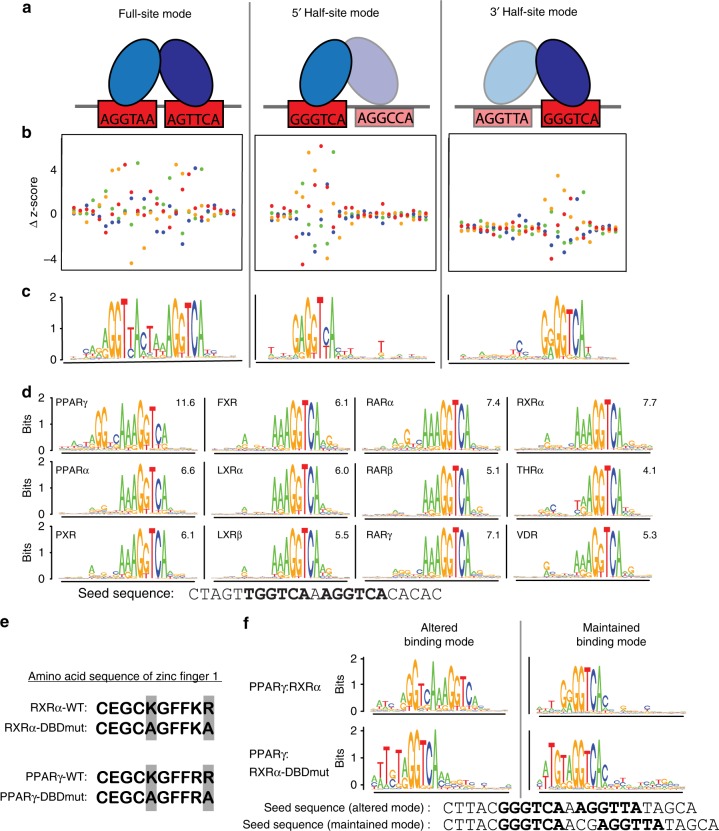
NR half-site binding mode. **a** Schematic of NR full-site or half-site binding modes. **b**, **c** For three seed sequences bound with different modes, the impact of SNVs on LXRα heterodimer binding and the corresponding DNA-binding logos are shown. Binding perturbation for each SNV is shown as a Δz-score from the median z-score of all four base variants at each position. Colors correspond to base identity indicated in logos below. **d** DNA-binding logos for all 12 NRs generated for the single DR1 seed sequence shown. **e** Amino acid sequence of zinc finger 1 for the wild-type RXRα, RXRα DNA-binding domain mutant, wild-type PPARγ, and the PPARγ DNA-binding domain mutant. Altered amino acids are highlighted in gray. **f** DNA-binding logos for individual seed sequences (shown) for which the binding mode was either altered (left) or maintained (right) for the PPARγ:RXRα-DNA binding domain mutant. Source data are provided as a Source Data file

We find that all NRs can bind with high affinity to half-sites (Fig. 2, final two columns). For all NRs, we obtain both 5’- and 3’-half-site logos, with the exception of PPARγ for which we only find clear 5’-half-site binding (Fig. 2). Half-site logos indicate that NR binding is only perturbed by SNVs in one half-site of a DR. To illustrate, we show the impact of SNVs on LXRα dimer binding to seed sequences with different binding modes (Fig. 3a–c, Supplementary Fig. 3). Critically, our data agree for PBMs probed with antibodies against either dimer member; therefore, half-sites are bound by NR heterodimers and are not a result of monomer binding. The presence of both full-site and half-site logos suggests that NRs can engage with DNA in two binding modes: (1) full-site mode where the NR engages with both half-sites and (2) half-site mode where the NR engages with a single half-site (either 5’ or 3’) (Fig. 3a).

To ensure that the widespread half-site binding was not a result of our methodology, we performed several analyses. First, we tested whether half-site binding was due to the orientation of the NR-binding site within the PBM probe with respect to the microarray slide. We find that regardless of orientation of the probe, binding mode is maintained (Supplementary Fig. 4). Second, we performed PBMs at successively lower concentrations to test whether half-site binding is affected by protein concentration and find nearly identical DNA binding logos at all concentrations (Supplementary Fig. 5). Finally, we used EMSA experiments to test the impact of base mutations on a DNA site bound in half-site mode (Fig. 1g, sequences P3, P3 5’-Abl, P3 3’-Abl). Critically, the 5’ half-site mode of PPARγ:RXRα determined by PBM is corroborated by EMSA experiments (i.e., 5’ half-site ablation greatly reduced binding whereas 3’ half-site ablation only modestly affected binding) (Fig. 1g, Supplementary Fig. 2). These results demonstrate that PBM-derived binding modes accurately represent native NR-binding modes.

NRs are known to bind half-sites (Fig. 1b), though half-sites have primarily been identified in ChIP-seq data and not through direct binding assays. Our analysis clarifies that NR heterodimers can bind half-sites, and can engage in a half-site mode even on canonical DR sites composed of two good half-sites (i.e., both half-sites score well using PWMs). For example, logos generated for a near-consensus DR1 seed sequence that scores highly by DR1 PWMs reveal both full- and half-site binding modes (Fig. 3d). While all NRs bind this site with high-affinity (z-scores are shown), only PPARγ binds in a full-site mode, while other NRs bind with nearly identical half-site modes. This shows that binding mode can vary for different NRs on the same DNA site, and that throughout the genome NR-binding to DR sites may in fact be mediated through a half-site binding mode.

### Role of monomers in half-site binding

To examine the contribution of each protein within an NR heterodimer to DNA binding, we created DNA-binding domain mutants (DBDmut) of RXRα and PPARγ. Two residues within zinc finger 1 of RXRα and PPARγ that make base-specific contact with DNA were mutated to alanines (K156A and R161A; and K132A and R137A, respectively, Fig. 3e)^31^. Binding of PPARγ:RXRα-DBDmut is highly correlated using either anti-RXRα or anti-PPARγ antibodies (R^2^ of antibody replicates given in Supplementary Data 1), showing that all DNA sites are bound by the mutant as a heterodimer. For PPARγ-DBDmut:RXRα, PBMs performed using an anti-RXR antibody are dominated by RXR homodimer signal, therefore binding of PPARγ-DBDmut:RXRα was determined using only the anti-PPARγ antibody. RXRα homodimer binding was not observed in wild-type heterodimer experiments (see above). All DBD mutant proteins were produced by IVT and PBM data for IVT-produced wild-type dimers agree with experiments using purified proteins, demonstrating that IVT proteins form heterodimers and function in DNA-binding assays similarly to purified proteins (Supplementary Data 4).

To confirm that these mutations abrogated DNA interactions, we examined the binding of mutant homodimers using PBMs. The mutant RXRα (RXRα-DBDmut) bound no sequences with z-score > 3.0 (as compared to a max z-score of 7.0 for PPARγ:RXRα-DBDmut described below), demonstrating an abrogation of sequence-specific DNA binding. The mutant PPARγ (PPARγ-DBDmut) showed binding with z-score > 3.0 to only five seed sequences. Previous experiments have shown residual DNA-binding activity for PPARγ DBD mutants^32^; therefore, we chose to disregard these five sequences from further analysis of the PPARγ-DBDmut:RXRα heterodimer experiments.

We first examined mutant heterodimer binding to sequences that PPARγ:RXRα binds in full-site mode. As expected, binding in full-site mode was almost completely abrogated for the PPARγ:RXRα-DBDmut (38/39 full-sites were lost). Of these sites, 40% (15/38) were now bound in the 5’ half-site mode (e.g., Fig. 3f and Supplementary Data 5), demonstrating an altered binding mode for the PPARγ:RXRα-DBDmut heterodimer. The remaining 60% (23/38) of these sites were bound with low affinity by PPARγ:RXRα-DBDmut, and scored below our z-score threshold for modeling interactions. The reciprocal mutant experiment with PPARγ-DBDmut:RXRα showed a complete loss of binding (i.e., z-score < 3.0) to nearly all of the full-sites (35/36, note that we have disregarded three sequences in this category as described above, Supplementary Data 5). These results demonstrate that DNA must be engaged by both dimer partners in order for PPARγ:RXRα to utilize a full-site binding mode, and shows that half-site binding can occur when only one partner can bind DNA.

Next, we examined which partner of the wild-type PPARγ:RXRα dimer engages with DNA when binding in a half-site mode. Of the 34 sequences that PPARγ:RXRα bound in a half-site mode, 53% (18/34) remained bound in half-site mode by PPARγ:RXRα-DBDmut, demonstrating that for these sequences PPARγ is making base-specific contacts with the DNA and can tolerate loss of base-specific DNA contacts mediated by RXRα (Fig. 3f). For the remaining 47% (16/34) of sequences bound by PPARγ:RXRα in a half-site mode, the mutant dimer binding was too low affinity to model (i.e., z-score < 3.0). Interestingly, PPARγ-DBDmut:RXRα, showed a loss of binding to 82% (29/32, note two sequences in this category were disregarded as above) of the half-site sequences. These results demonstrate that a single partner of an NR heterodimer can mediate half-site binding; however, for other sites, mutation of either NR partner can lead to loss of heterodimer binding. The strong impact of mutations to either member of the heterodimer may be attributable to the ability of either partner to engage with the half-site, or to a contribution in binding energy through non-specific interactions from the non-engaged partner, which were abrogated by the mutations we made.

### NR spacer preferences do not define high-affinity binding

**Fig. 4:**
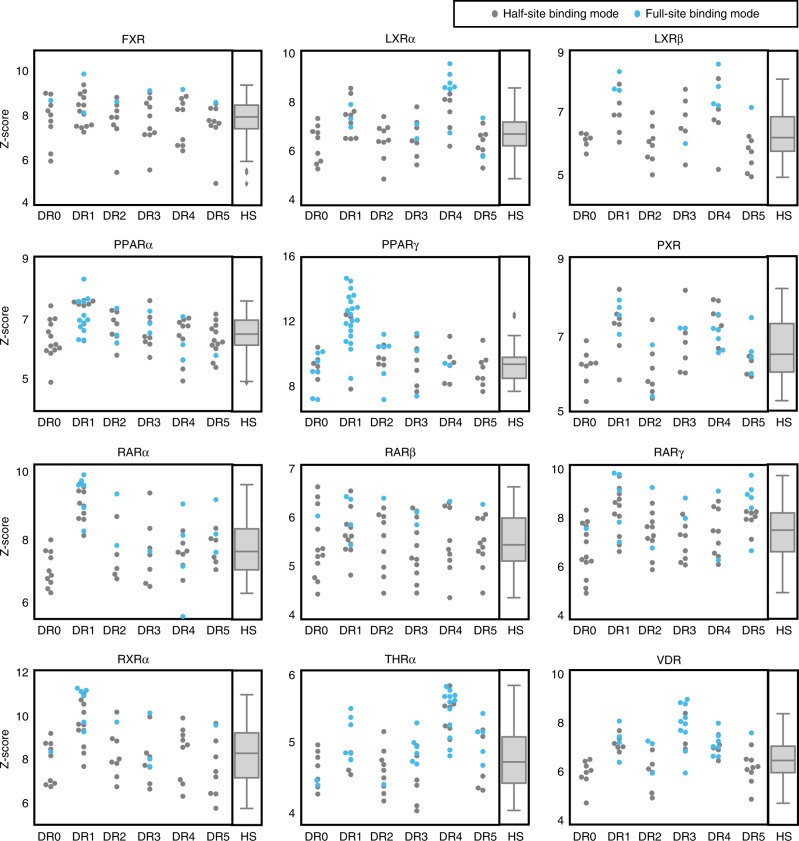
NR-binding affinity and mode for sequences at each DR spacer length. At each spacer length, the replicate averaged z-score of the highest scoring SNV for each seed sequence is shown; seed sequences with z-score < 3 are not represented. Colors indicate binding mode for each seed sequence. For each NR, box plots show the z-score distributions for all sites that are bound in half-site modes across all direct repeat spacer lengths (the aggregate of all gray dots). Center line: median; box limits: upper and lower quartiles; whiskers: last datum within 1.5x interquartile range. Source data are provided as a Source Data file

Previous studies have examined the impact of DR spacer length on NR binding^2,8,10,12^; however, our results show that NRs can bind in a half-site mode even on DR sites, which complicates the interpretation of these experiments. SNV binding models are advantageous as they allow examination of NR-binding mode on each sequence, thus facilitating a more rigorous assessment of NR spacer preferences. We analyzed the NR-binding landscape to all 24 seed sequences at each DR spacing and used the resulting binding logos to annotate whether each sequence was bound in a full-site or half-site mode (Fig. 4).

In contrast to the prevailing view of NR spacer preferences^2,8,22,33^, we observed that NRs can bind with high affinity to DRs at all spacer lengths (Fig. 4). For most NRs, high-affinity binding to many DR spacer lengths is predominantly mediated via a half-site binding mode (Fig. 4 gray dots). Despite this promiscuous NR binding, our results recapitulate literature-reported NR spacer preferences, which are demonstrated by an enrichment of full-site binding mode and higher z-scores for specific DR spacer lengths (Fig. 4 blue dots). For example, PPARγ engages with DR1 sequences almost entirely via a full-site binding mode. Similar observations corroborate previously described DR-spacing preferences, for example LXRs (DR1 & DR4), THRα (DR4), and VDR (DR3) (see Fig. 1b). However, for most NRs the increase in binding affinity to certain DR spacers is more modest than observed for PPARγ, suggesting that spacer preferences do not define the DNA binding landscape of each NR. In fact, for some NRs the canonical DR-spacing preferences appear primarily as enrichment in full-site binding mode, but not a large increase in binding affinity. For example, PPARα preferentially engages with DR1 sites in a full-site binding mode but only binds with moderately higher z-scores to these sites. Our results reveal a complicated NR-DNA binding landscape in which DR spacer preferences contribute to altered NR-binding modes and binding affinity, but which do not strongly define the landscape of all possible high-affinity binding.

### Diverse mechanisms contribute to NR-DNA binding

**Fig. 5:**
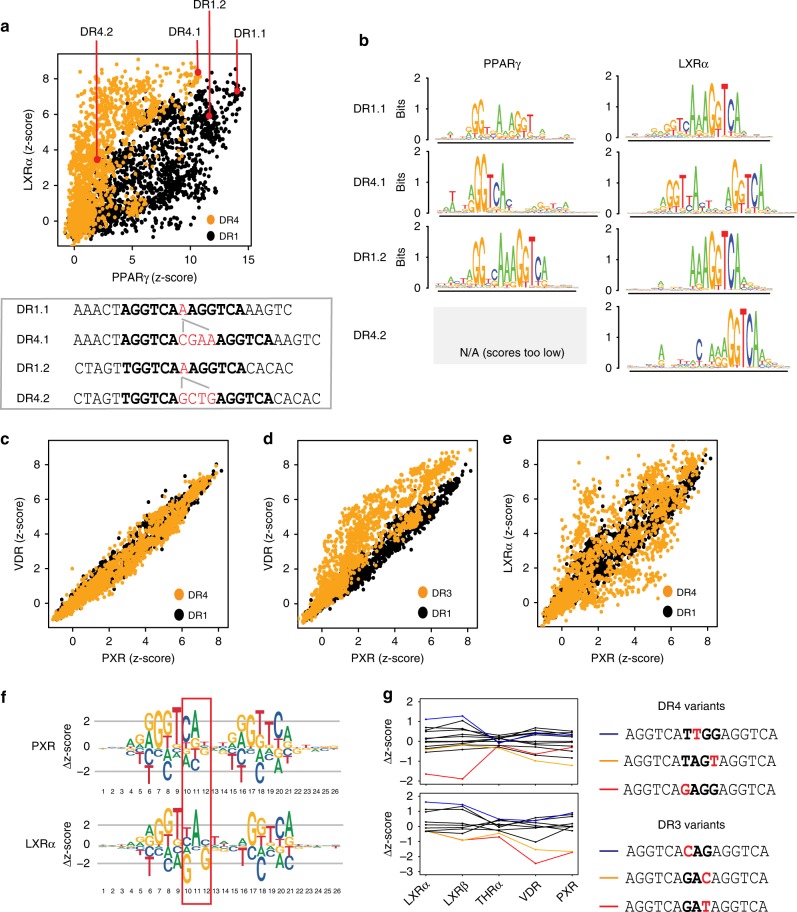
NR specificity differences. **a** Scatter plots of LXRα and PPARγ binding to DR1 and DR4 sites. Each spot is the average of ∼5 replicates for each unique DNA sequence (∼1600 at each spacer length) on the PBM. DR1 and DR4 spacer-variant sequences are shown in the box below panel. **b** Binding logos generated for LXRα and PPARγ for the spacer-variant seed sequences from **a** are shown. **c** Scatter plots as in **5a** of VDR and PXR binding to DR1 and DR4 sites. **d** Scatter plots as in **5a** of VDR and PXR binding to DR1 and DR3 sites. **e** Scatter plots as in **5a** of LXRα and PXR binding to DR1 and DR4 sites. **f** DR4 z-score logos, directly representing Δz-scores of SNV binding, are shown for LXRα and PXR. Δz-scores are calculated (separately for each NR) as the difference from the median of all SNV variants. **g** Differential binding of NRs to spacer-sequence variants. (Top panel) Binding is shown for five NRs to the DR4 seed sequence 5’-AGGTCATAGGAGGTCA-3’ and all 12 SNVs of the spacer region (spacer region in bold). Δz-scores are calculated as in **5f**. (Bottom panel) Binding is shown for five NRs to the DR3 sequence 5’-AGGTCAGAGAGGTCA-3’ and all nine corresponding SNVs of the spacer region (spacer region in bold). Examples of highly variant spacer sequences are indicated. Source data are provided as a Source Data file

Despite broad similarities seen in binding logos (Fig. 2), our dataset reveals that NR-binding differences result from multiple mechanisms: DR-spacing preferences, DNA-base preferences, and DNA-binding-mode differences. To illustrate the roles of spacing preferences and binding modes, we compared the binding of PPARγ and LXRα to DR1 and DR4 sites and observe both NR-shared and NR-specific binding sites (Fig. 5a). The LXRα preference for DR4 sites and PPARγ preference for DR1 sites are demonstrated as biases in the z-score distributions. However, as we see high-affinity binding of PPARγ to DR4 sites and LXRα to DR1 sites, the aforementioned preferences do not explain all high-affinity binding. To explicitly test the impact of DR spacing, we examined binding to pairs of seed sequences that differ only in their spacer length (e.g., Fig. 5a, sequences DR1.1 and DR4.1). Critically, we examined the DNA-binding mode for each interaction using the DNA-binding logos generated for each seed sequence (Fig. 5b). For PPARγ, DR4 sites are bound with lower affinity than corresponding DR1 variants; however, DR4.1 is still bound with high affinity via a half-site binding mode (Fig. 5a). In contrast, when LXRα binds via a full-site mode the DR4 variant is bound with higher affinity (DR1.1 vs DR4.1), but when binding via a half-site mode the DR4 variant is bound with lower affinity (DR1.2 vs DR4.2) (Fig. 5a). Therefore, both NRs can bind the same sequence with high affinity, but may utilize distinct binding modes. Taken together, these results demonstrate that both spacer preference and binding mode contribute to binding specificity.

To investigate the plasticity of DR spacer preferences, we compared PXR and VDR, which exhibit broadly similar binding to DR1 and DR4 sites but differ for DR3 binding. PXR and VDR bind with nearly identical specificity to DR1 and DR4 sites (Fig. 5c, R^2^ = 0.98 for both); however, the VDR preference for DR3 sites is seen as an increase in z-score for most DR3 sequences (Fig. 5d). This example illustrates that NRs can bind similarly on one DR spacing while having distinct binding preferences for another DR spacing.

Next, we asked whether shared spacer preferences might constrain DNA-base preferences. PXR and LXRα both exhibit preferences for DR1 and DR4 sites (Fig. 4); their binding profiles are highly correlated for DR1 sites (R^2^ = 0.95), but show lower correlation on DR4 sites (R^2^ = 0.83) (Fig. 5e). Analysis of the standard DNA-binding logos did not reveal a strong basis for differential DNA-base preferences. However, by directly examining the impact of SNVs on binding via visualization as an energy matrix (which indicates both favorable and unfavorable interactions), we see strong differences between PXR and LXRα at positions 10 and 12 (Fig. 5f). The majority of the PXR-specific binding sites are explained by the existence of a guanine base at position 10 that is highly disfavored by LXRα (G10 carries a z-score penalty of −3.21 for LXRα compared to −0.47 for PXR). We note that the highly unfavorable G10 preference for DR4 sites (Δz-score = −3.21) is not observed for DR1 sites (Δz-score = −0.65), demonstrating that this NR-specific preference is not shared across all spacer lengths (Supplementary Fig. 6). These results highlight the advantages of visualization of energy logos over traditional DNA binding logos, and demonstrate that novel base preferences can arise on DR sites of different lengths.

In NR-binding logos, we observe base preferences in the spacer sequence between DR half-sites (e.g., Figs. 2, 5f, positions 12–15). We note a strong preference for an adenine in the spacer of DR1 sites, which has been demonstrated for PPAR and other NRs^34^; however, such a distinct base preference is absent at longer spacer lengths (DR2–DR5). To investigate the contribution of the spacer sequence to NR specificity, we examined how spacer variants modulate NR-DNA binding (Fig. 5g). We focused our analyses on NRs that exhibit preferences for DR3 and DR4 sites. Examining the binding affinity distribution for SNVs within the spacer of a single seed sequence, we find that the spacer sequence can have considerable impact on binding affinity in an NR-specific manner (Fig. 5g), consistent with reports that NRs make DNA contacts with the spacer sequence^35^. Given the established role for DNA shape in TF binding specificity^36–38^, we investigated whether DNA shape features in the spacer sequence might also contribute to the selectivity for different binding sites. We examined DNA shape features for spacer variants of DR3 and DR4 sites that enhance or diminish the binding of LXRα and VDR (Supplementary Fig. 7). The DNA shape features (i.e., major groove width, helix twist, propeller twist, and roll) examined are nearly identical for all comparisons. However, we observed a significant difference in the major groove width and roll parameters for VDR binding to DR3 sites. Our results suggest that DNA shape may also play a role in NR-binding specificity. Future studies that more exhaustively sample spacer sequences may enable identification of more subtle differences.

### Genomic binding agrees with in vitro binding preferences

**Fig. 6:**
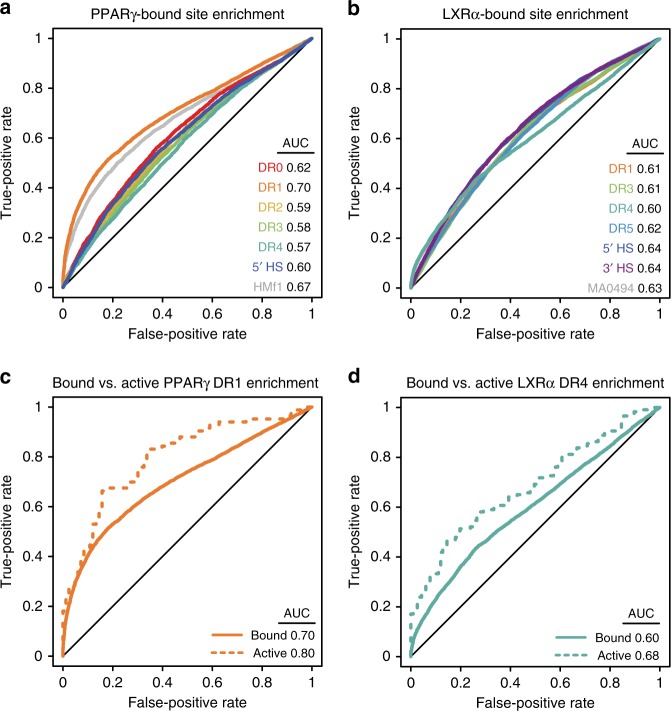
Genomic enrichment of NR-binding motifs. **a** Receiver-operating characteristic (ROC) curves for PPARγ motif enrichment in ChIP-seq data. ROC curves and area under the curve (AUC) values for different PBM-derived NR-binding models are shown, along with the results for best-performing published PPARγ DR1 motif (HOCOMOCO-f1, HMf1^28^). Motif enrichment for all models had *p*-values < 10^−46^, using a Wilcoxon rank sum test with continuity correction and Bonferroni corrected for multiple hypotheses. **b** ROC curves for LXRα motif enrichment in ChIP-seq data. ROC curves and AUC values for different LXRα binding models are shown. Results for best-performing published LXRα DR4 motif (JASPAR MA0494.1^29^) are shown. **c** ROC curves and AUC values are shown for PPARγ DR1 motif enrichment in reproducibly-bound PPARγ ChIP-seq peaks (solid lines, Methods), and for those peaks occurring within 10 kb upstream of differentially expressed genes (i.e., active peaks). **d** ROC curves and AUC values are shown for LXRα DR4 motif enrichment in reproducibly-bound LXRα ChIP-seq peaks (solid lines, Methods), and for those peaks occurring within 10-kb upstream of differentially expressed genes (i.e., active peaks). Source data are provided as a Source Data file

Our NR-binding landscape (Fig. 2) indicates DNA binding to DR sites with many spacer lengths. To determine whether NRs use these diverse sites in vivo, we evaluated the ability of our PBM-derived models to explain in vivo-bound regions from published ChIP-seq datasets (Methods). Examining published PPARγ binding data in HT29 colorectal cancer cells (GSE77039)^16^, we find that all PPARγ models (DRs and half-sites) can discriminate bound regions from unbound. However, the DR1 model best describes the data (area under the curve (AUC) = 0.70, Fig. 6a), in agreement with established PPARγ binding preferences and our PBM data (Fig. 4). Testing other published DR1 models^28–30,39^ (Methods and Supplementary Data 3), we find the HOCOMOCO-f1 DR1 model performs best (AUC = 0.67) and with similar accuracy to our DR1 model. These results suggest that binding to DR1 sites is an important determinant of in vivo PPARγ binding. In contrast, all models for LXRα yield similar AUCs (Fig. 6b), with the canonically preferred DR4 model performing similarly to the half-site models. Testing other published DR4 models we find JASPAR MA0494.1 (DR4) performs the best (AUC = 0.63), and performs similarly to PBM-derived half-site models (AUCs = 0.64). These in vivo binding results are consistent with our in vitro binding data, which show a strong DR1 preference for PPARγ and broader binding preferences for LXRα.

### Functional sites agree with canonical NR preferences

We hypothesized that functional binding sites that regulate gene expression may have a different motif composition than the full set of genomic binding sites. Binding sites were annotated as ‘functional’ if they were located within 10 kb upstream of the transcription start site of genes whose expression changed >2-fold upon agonist treatment (GSE77039^16^, Methods). We then performed motif enrichment analysis for these functional PPARγ or LXRα binding sites. Strikingly, we observe an increase in the enrichment of the PPARγ DR1 and the LXRα DR4 models for their respective functional sites (Fig. 6c, d). These same trends are observed when we use alternate genomic constraints to define functional sites (i.e., 10 kb up- and downstream, or 50 kb upstream) (Supplementary Fig. 8). These results are consistent with a model wherein NRs preferentially utilize DR full-sites at a canonical spacing for activating transcription, while genome-wide binding is determined by a broader set of DR and half-site sequences, consistent with our in vitro binding data.

### NRs binding via a half-site mode can drive gene expression

**Fig. 7:**
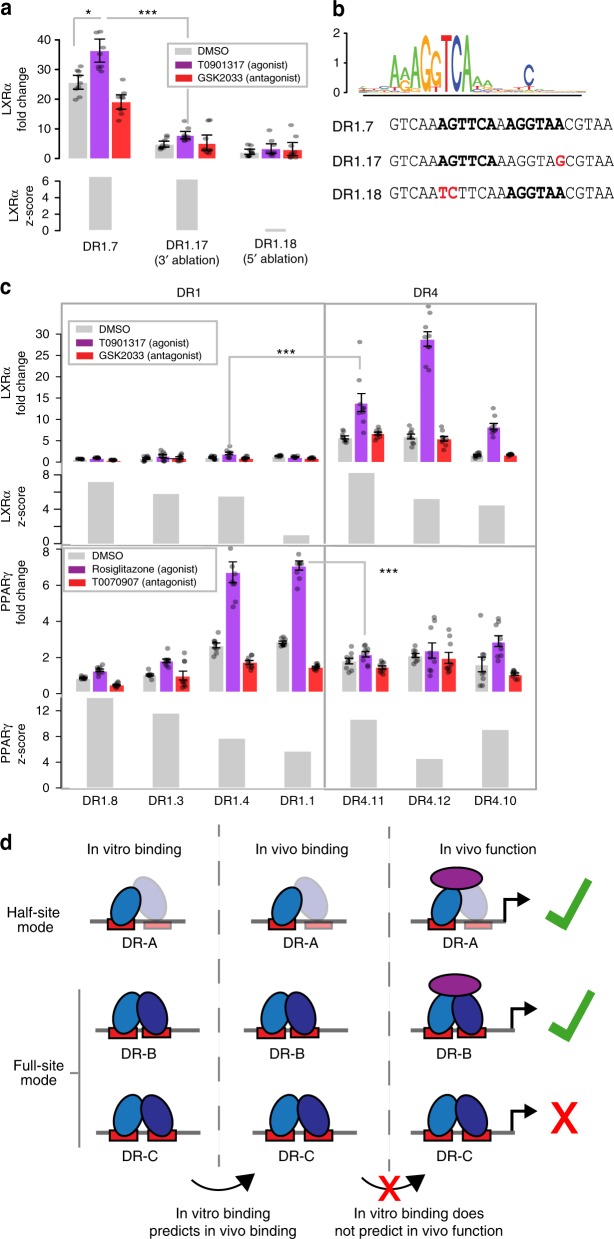
Activity versus affinity for distinct classes of NR-binding sites. **a** LXRα-dependent activity and binding affinity of a sequence bound in a half-site mode. Luciferase reporter gene activation, and corresponding z-scores, are shown for the DR1.7 sequence, which is bound in a half-site mode on PBM, and sequences with each half-site ablated (DR1.17 and DR1.18), sequences shown in **b**. Fold-change reporter expression indicates luciferase activity in HEK293T cells over-expressing LXRα and RXRα normalized to cells not over-expressing these proteins. Fold-change expression is shown for cells treated with DMSO (vehicle), agonist (T0901317), or antagonist (GSK2033), and values represent mean over nine replicate measurements (error bars = SEM). Reporter gene *p*-values: * < 0.01, *** < 0.0001 (calculated using Student’s two-tailed *t*-test). **b** Logo for LXRα heterodimer binding to DR1.7, and sequences for DR1.7, DR1.17, and DR1.18 discussed in **a**. **c** LXRα- and PPARγ-dependent activity and PBM-derived binding scores to select DR1 and DR4 sites. Fold-change expression for LXRα is as described in **a**. Fold-change for PPARγ is shown for cells treated with DMSO (vehicle), agonist (rosiglitazone), or antagonist (T0070907), and values represent the mean over nine replicate measurements (error bars = SEM). **d** Overview of relation between NR in vitro binding, in vivo binding, and function. Source data are provided as a Source Data file

Our analyses reveal widespread binding of NRs to half-site sequences both in vitro and in vivo. Furthermore, we show that half-site mode is utilized by NRs to bind not only to half-sites, but also to canonical DR sites. To determine whether NR half-site mode binding is functional and can drive gene expression, we assayed the ability of LXRα to activate a reporter gene from a binding site bound in a half-site mode on our PBM. Expression of luciferase reporter genes was monitored in HEK293T cells in the presence of over-expressed LXRα:RXRα and ligand or vehicle (Methods). We find that LXRα strongly induces gene expression, in a ligand-dependent manner, from a DR1 site (DR1.7) that is bound in a half-site mode by PBM (Fig. 7a, b, logo illustrates the 5’-half-site binding mode). Ablating the 5’ half-site sequence (DR1.18) abrogates binding and drastically reduced reporter gene expression. Ablating the 3’ half-site (DR1.17) does not affect binding affinity; however, unexpectedly, it strongly affected reporter gene expression, demonstrating that in vitro affinity does not necessarily predict binding-site activity. Therefore, NRs binding via a half-site mode in vitro can drive gene expression, but DNA bases that do not affect binding affinity in vitro can affect function in vivo.

### NR spacing preferences are defined by function not affinity

We next examined the ability of LXRα and PPARγ to promote gene expression from a range of DR1 and DR4 binding sites (Fig. 7c). In general, PPARγ drives higher levels of gene expression from DR1 sites, and LXRα functions better on DR4 sites, in agreement with their canonical spacer preferences. However, we see exceptions to these simple rules. First, LXRα can promote expression from the DR1.7 site (Fig. 7a) at a comparable or higher level than from the three DR4 sites (Fig. 7c). Second, for PPARγ, several high-affinity DR1 sites (DR1.8, DR1.3) show comparable or lower activity than the three DR4 sites, which are all bound with comparable or lower affinity. Complicating the interpretation, without NR overexpression, the DR4 sites exhibit lower reporter gene activity than DR1 sites (Supplementary Fig. 9). This low basal activity may exaggerate the NR-dependent activation determined for these sites, which is calculated as the fold-change between basal and NR-over-expressed conditions. Despite these complications, it is clear that affinity does not strongly predict activity of different NRs.

## Discussion

Here we report the most comprehensive DNA binding dataset to date for the type II NRs, and provide a revised framework for interpreting NR-binding and regulatory specificity. We demonstrate more promiscuous DNA binding for NRs than has been previously reported, challenging the view that NR-binding specificity is defined solely by distinct DR spacer preferences. Our findings agree with other PBM-based studies of NR homodimers that demonstrated nearly identical binding for RXRα and COUP-TF2^40^, and found that NR specificity does not solely depend on DR-spacing rules^34,40^. We demonstrate that NR-binding-site activity does not follow binding affinity, and that the canonical NR DR spacer-length preferences better reflect activity rather than DNA-binding-site affinity. Our revised framework for NR-binding and function shows that NRs bind DNA via two binding modes to a broad set of DR and half-site sequences; this binding corresponds with in vivo binding, but does not correspond to in vivo function, which may involve additional layers of specificity (e.g., allostery) (Fig. 7d). Future studies that focus on refining the rules for NR-binding-site activity will clarify this general framework and improve genomic analyses aimed at predicting NR-dependent gene regulation, or the impact of SNPs on NR function, as in a recent analysis of PPARγ function^18^.

Our study challenges the prevailing view that each NR heterodimer prefers binding to DR sites of specific spacer lengths^2,8^. We show that all NRs can bind with high affinity to many DR spacer lengths in a full-site binding mode. Previous studies that sought to identify DR spacer preferences did not explicitly account for multiple NR-binding modes, potentially complicating their interpretations^2,8,10,12^. While we observe previously described DR spacer preferences, our study suggests a distinct biophysical interpretation for these preferences. We propose that DR preferences of NRs are not based on a large increase in binding affinity, but arise from a preference to bind in a full-site mode over a half-site mode, coupled with a moderate increase in affinity (i.e., LXRα and PPARα, Fig. 4). The implication that NR spacer preferences are primarily about binding mode, rather than affinity, may provide a biophysical interpretation of NR preferences that links binding mode to in vivo function.

The disagreement between the promiscuous NR binding seen in our study and the canonical DR spacer preferences reported in the literature may be explained by differences in the approaches utilized. DR spacer preferences were initially characterized on a small number of DNA sequences obtained from promoter regions of genes that were upregulated upon ligand treatment, naturally biasing towards functional genomic binding sites^2,8,10,12^. Other high-throughput methodologies used to examine NR heterodimer binding preferences bias towards high-affinity binding sites and thus do not capture the full landscape of NR-binding specificity^30^. Our PBM approach, which queried the binding across a broad range of affinities and DR spacer lengths, reveals a more promiscuous NR-binding landscape.

Our NR-binding data are consistent with in vivo binding, and provide an updated framework for interpreting genome-wide binding data. For example, PPARγ ChIP-seq peaks are best modeled by a DR1 motif, consistent with the high-affinity binding observed for DR1 sites. In contrast, LXRα ChIP-seq peaks are modeled equally well by most DR models and half-sites (Fig. 6), consistent with broader in vitro specificity for LXRα. We note that a DR4 motif was identified by de novo motif analysis using this LXRα ChIP-seq dataset^16^, but only when restricting the analysis to the highest scoring ChIP-seq peaks; when motif finding is performed on the full dataset, a half-site motif is identified. This example illustrates a source of confusion in the field: reinforcement of established NR-binding preferences by conclusions supported by only a small fraction of the genome-wide binding data.^13,16,41^. Re-interpreting the genomic data in light of our dataset, we find that the broader specificity found in vitro is consistent with in vivo binding.

Unexpectedly, we found that all type II NR heterodimers have the ability to bind DNA via a half-site mode on both full-sites and half-sites. This is a clear example of DNA-based allostery, in which interactions with DNA alter the structure of DNA-bound TFs. Allostery has been reported for the NRs^42–47^, and provides a mechanism to decouple affinity and activity. A provocative idea is that NR-binding mode may predict activity and explain NR functional preferences. Supporting this idea, a recent study of the glucocorticoid receptor (GR), a steroid hormone nuclear receptor, showed that GR homodimers can bind to half-site sequences in vivo to repress gene expression^48^. Our data on the preference of PPARγ and LXRα to bind in a full-site mode and drive gene expression from DR1 and DR4 sites, respectively, offer additional support for this idea. Other work has demonstrated that NR binding can be altered by cofactor proteins^49,50^, raising the possibility that NR binding modes may be altered in the presence of endogenous cofactors. Future studies that assess NR-DNA binding and binding modes in the presence of cofactors will help clarify the relationship between NR-binding mode, affinity, and activity. Our PBM dataset provides a valuable resource for these future studies aimed at elucidating the mechanisms of NR specificity in gene regulation.

## Methods

### Protein expression and purification

Full-length, wild-type human RXRα and PPARγ isoform 1 constructs were cloned into the Gateway vector pDEST17 (LifeTech) for propagation, mutagenesis, and expression. A TEV-protease recognition sequence was included between the coding sequence of the His-tag and RXRα and used to cleave the His-tag after purification. His-tagged RXRα and PPARγ were expressed using the BL21(DE3) *E. coli* strain (NEB). Transformed bacteria were propagated on Luria-Bertani broth (LB) plates supplemented with 100 μg/mL of carbenicillin. Protein expression was carried out in LB supplemented with 100 μg/mL of carbenicillin, with an initial outgrowth at 37 °C up to an OD of 0.4, transferred to ∼20 °C until they reached an OD of 0.6–0.7 and then induced with 1 mM IPTG. Protein was expressed at room temperature (∼20 °C) for 3 h. Cells were pelleted and stored at −80 °C until purification. Purification was carried out using HisTrapFF columns (GE Healthcare). The binding buffer was composed of 20 mM Tris HCl, 300 mM NaCl, 25 mM Imidazole, and 1 mM DTT and the elution buffer was composed of 20 mM Tris HCl, 300 mM NaCl, 250 mM Imidazole, and 1 mM DTT. Buffers were supplemented with cOmplete Mini protease inhibitor tablets according to the manufacturer’s instructions (Roche). Eluted fractions were analyzed by SDS-PAGE and fractions containing protein were combined. For PPARγ, the combined elution fractions were buffer exchanged into phosphate buffered saline pH 7.4 with 1 mM PMSF and 10% glycerol using an Amicon Ultra centrifugal filter (30k MWCO). Elution fractions of RXRα were dialyzed against three changes of binding buffer. Next, the His-tag was cleaved from RXRα by overnight incubation at 4 °C with TEV protease (Sigma–Aldrich). After cleavage, the RXRα sample was re-purified as described above; however, this time the flow-through fraction from the column loading was collected and used in all PBM experiments, as this fraction contained the RXRα from which the His-tag was successfully cleaved. The combined flow-through fractions were buffer exchanged into phosphate buffered saline pH 7.4 with 1 mM PMSF and 10% glycerol using an Amicon Ultra centrifugal filter (30k MWCO).

The RXRα and PPARγ DNA binding domain mutants were made by site-directed mutagenesis using the NEB Q5 site-directed mutagenesis kit (New England Biolabs) following the manufacturer’s instructions. Primers used for the mutagenesis were: RXRα: Forward = 5’-CTTCTTCTTCAAGGCGACGGTGCGCAAGGACCTG, Reverse = 5’- CCCGCGCACCCCTCGCAGCTGTACACTCCATCAGC; PPARγ: Forward = 5’-CTTCCGGGCAACAATCAGATTGAAGCTTATCTATGACAG, Reverse = 5’- AAACCCGCGCATCCTTCACAAGCATGAACTCCATAGTG. For DNA binding domain mutant experiments, both wild-type and mutant RXRα were expressed using the PURExpress IVT kit (NEB) according to manufacturer instructions. The concentration of all IVT-produced proteins was estimated by western blot by comparison to purified proteins. All other purified proteins used were purchased (see Supplementary Data 1 for details).

### PBM design

PBM experiments were performed using custom-designed microarrays (Agilent Technologies Inc. AMADID 084387, 4 × 180 K format). PBM probes contain a 24 nt constant primer region, a 34 nt variable region, and a 5’ GC dinucleotide cap (Supplementary Data 4). For each unique SNV probe sequence, five replicate probes were included in each orientation (10 probes per unique sequence). For all other probe sequences four replicate probes were included with the 34 nt variable region in each orientation (8 probes per unique sequence).

*SNV probes*: DR seed sequences, defined by two 6-bp half-sites and a variable spacer (0–5 bp), were aligned within in the 34 nt variable region of each PBM probe. For each seed sequence, SNV probes were created that had a single-nucleotide variant at each position of the DR half-sites, the spacer sequence between the DR half-sites, and in the 5 bp flanks of each site. Therefore, for a single 13 bp DR1 site (i.e., 6 + 6 + 1 = 13), including 5 bp flanks on either side, there would be 69 (i.e., 23 × 3) unique SNV probe sequences.

*Half-site ablation probes*: For each DR seed sequence, probe variants were created with each half-site ablated. Ablations were performed by identifying the position in the half-site that contributes most to the NR-binding score and replacing it with a penalizing base.

*Random genomic probes*: 34 nt regions were randomly chosen from the UCSC hg19 build of human genome. Sequences were removed that contained Ns or single-nucleotide repeats longer than three nucleotides.

### PBM experiments and analysis

Microarrays were double-stranded as previously described (PBM double-stranding primer 5’-CCTTCATTCTACGCTGTCAATCGC-3’)^21,51^. All washes were performed in coplin jars on an orbital shaker at 125 rpm. Double-stranded microarrays were first pre-wetted in PBS containing 0.01% Triton X-100 for 5 min, rinsed in a PBS bath, and then blocked with 2% milk in PBS for 1 h. After blocking, arrays were washed in PBS containing 0.1% Tween-20 for 5 min, then in PBS containing 0.01% Triton X-100 for 2 min and then rinsed in a PBS bath. Proteins were then incubated on the array for 1 h in a binding reaction containing: PBS pH 7.4 with 2% milk, 0.02% Triton X-100, 1 mM DTT, 0.2 mg/mL bovine serum albumin, and 0.4 mg/ml salmon testes DNA (Sigma D7656). See Supplementary Data 1 for protein concentrations. Preliminary PBM experiments for PPARγ:RXRα and RXRα were performed with and without the ligands rosiglitazone and 9-*cis* retinoic acid, respectively, and we found no change in NR binding; therefore, all experiments were performed in the absence of ligand. Following the protein incubation, microarrays were washed with PBS containing 0.5% Tween-20 for 3 min, then in PBS containing 0.01% Triton X-100 for 2 min followed by a brief PBS rinse. Microarrays were then incubated with 20 μg/ml of primary antibody in 2% milk in PBS for 20 min. For heterodimers, separate experiments were performed using an antibody against each protein within the heterodimer. In all experiments, anti-RXRα antibody (Active Motif 61029) was used to detect RXRα and anti-His antibody (Sigma H1029) was used to detect the NR partner with the following exceptions: anti-PPARγ antibody (Abcam 41928) was used in all experiments with PPARγ, and Alexa488-conjugated anti-GST antibody (Life Tech A11131) was used for all PPARα experiments. Excess primary antibody was removed by washing with PBS containing 0.05% Tween-20 for 3 min and then in PBS containing 0.01% Triton X-100 for 2 min. Arrays were next incubated with 20 μg/ml of Alexa488-conjugated secondary antibody (anti-mouse A488, Life Tech A11001) in 2% milk in PBS for 20 min (PPARα was probed with an Alexa488-conjugated anti-GST primary antibody as described above and did not require a secondary antibody). Excess antibody was removed by washing 2x with PBS containing 0.05% Tween-20 for 3 min and then in PBS for 2 min. Microarrays were scanned with a GenePix 4400 A scanner and fluorescence was quantified using GenePix Pro 7.2. Exported data were normalized using MicroArray LINEar Regression^21^. Microarray probe sequences and fluorescence values from each experiment are provided (Supplementary Data 4). NR dimers exhibit an orientation-specific bias in our PBM experiments; therefore, data from probes in a single orientation (i.e., ‘_o1′ probes in Supplementary Data 4 was used in our final analysis. However, all results were observed for probes in both orientations and models from each orientation showed good agreement.

Position frequency matrices (PFMs) and DNA-binding logos were generated for each seed sequence with z-score >3.0 using the previously described SNV-based approach^52^, with β set to 15/maximum z-score. Briefly, logos for single seed sequences are generated using the binding data to each seed sequence and all the single-nucleotide variant (SNV) sequences for that seed sequence. For a binding site of length L there will be 3xL SNV sequences. Logos for an NR binding to a specific DR spacer length are determined by averaging over the individual seed sequence logos. To generate logos for a specific DR spacer length (Fig. 2), PFMs for all seed sequences at that spacer length were clustered into full-site, 5’-half-site or 3’-half-site PFMs. Average PFMs of each type (i.e., full, 5’-half-site or 3’-half-site) were then generated by directly averaging over the individual PFMs (i.e., averaging individual matrix elements and normalizing each column to 1). As the half-site PFMs are the same length regardless of the starting DR seed length, the final 5’-half-site and 3’-half-site PFMs were further averaged over PFMs generated at all spacer lengths. The z-score energy matrix (Fig. 5f) was generated in the same manner, without the initial transformation from z-score to frequency^52^.

### Reporter gene assays

PPARγ, LXRα, and RXRα were cloned into the N-terminal His-tagged protein mammalian expression plasmids (pDEST26, LifeTech). Reporter constructs for test sequences were ordered synthesized (Twist Bioscience) and were flanked by two BsaI cut sites, which were used to clone the sequences into pNL3.1-minP/Nluc (Promega). All sequences tested can be found in Supplemental Data 2. HEK293T (ATCC) cells were cultured in DMEM (Gibco 11965-092) supplemented with 10% FBS (Gibco 26140079). Cells were plated in tissue culture treated 96-well plates seeded at a density of 12,500 cells per well and allowed to adhere overnight. PEI:DNA complexation reactions were prepared at a ratio of 3:1 (PEI:DNA) in 500 μl of Opti-MEM (Gibco 51985-034) and allowed to complex for 20 min at room temperature. Each 96-well plate well received 20 μl of transfection mixture containing 16 ng of total plasmid: 1 ng of transfection normalization plasmid (pGL4.54-Luc2/TK); 10 ng of reporter plasmid (pNL3.1-minP/Nluc); and either 5 ng of empty pDEST26 for the no overexpression conditions (NoOE); or 2.5 ng of RXRα in pDEST26 combined with either 2.5 ng of PPARγ or LXRα in pDEST26 for protein overexpression condition (OE); Twenty-four hours after transfection, 80 μl of media was removed from each well and replaced with 80 μl of fresh media containing the appropriate ligand treatment. PPARγ ligands were 1 μM rosiglitazone (Sigma–Aldrich) and 1 μM T0070907 (Sigma–Aldrich). LXRα ligands used were 1 μM GSK2033 (Sigma–Aldrich) and 500 nM T0901317 (Sigma–Aldrich). Luciferase activity was assessed 18 h after addition of the ligand using the Nano-Glo Dual Luciferase reporter assay system (Promega). Dual luciferase signal was quantified using a VICTOR-3 plate reader (PerkinElmer). To control for transfection efficiency, the Nluc reporter plasmid signal was normalized to the constitutive luciferase signal (i.e., signal from pGL4.54 plasmid) (Nluc/Luc2). Normalized signal for all test DNA elements were then further normalized to empty vector (pNL3.1-Nluc with an insert of equal length to test sequences but lacking any half-site or direct repeat sequences). Fold-induction values for each protein + reporter combination were calculated relative to the background activity of each reporter plasmid in the absence of protein overexpression: (protein + reporter)/(control + reporter) = OE/NoOE (Supplementary Fig. 9). Reporter assays were performed as three biological replicates with three technical replicates per biological replicate.

### EMSA experiments

Complementary DNA oligonucleotides (sequences in Supplementary Data 2) were ordered from IDT and annealed in a thermocycler by raising the temperature to 98 °C and reducing the temperature by 0.1 °C/sec until a temperature of 4 °C was reached. All DNA sequences are provided in Supplementary Data 2. EMSA buffer formulation for all reactions was 1x PBS with 0.2% BSA, 5 mM DTT, 10% glycerol, and 0.02% Triton-X100. For the direct binding experiment, 1 nM of IR700-labeled P1 probe was incubated with varying concentrations of PPARγ:RXRα in a 20 μL reaction. For competition experiments, 2 nM of IR700-labeled P1 probe was incubated with PPARγ:RXRα (12 nM:4 nM) in a 20 μL reaction with various concentrations of unlabeled competitor sequences (0, 0.2, 0.63, 2, 6.3, 20, 63, 200, 630, and 2000 nM). Reactions were incubated for 1 h at room temperature and then run in 0.5x TBE on a 6% TBE-acrylamide gel at 50 V for 3 h. Gels were scanned on the Odyssey CL-X (LI-COR) at 84 μM resolution. Fluorescence of the shifted band was quantified using ImageStudioLite software. All K_d_ calculations were done with DynaFit 4 software^53^ using a previously described competition protocol^54^. Percent competition was calculated by the formula:

% inhibition = (*F*_*0*_- *F*_*c*_)/*F*_0_*100

*F*_*0*_: fluorescence of shifted band with no competitor DNA

*F*_*c*_: fluorescence of shifted band at given concentration of competitor DNA.

### Enrichment of NR-binding sites in ChIP-seq data

Receiver-operating characteristic (ROC) curve analyses were performed to quantify the extent to which NR-bound (true positive) regions scored more highly than unbound (true negative) regions with PWM models. True-positive regions for LXRα and PPARγ were derived from ChIP-seq data from HT29 colorectal cancer cells (GSE77039)^16^. ChIP-seq was available for two biological replicates of HT29 cells treated with agonist (GW3965 for LXRα or rosiglitazone for PPARγ) for 2 h and 48 h. For each NR, ChIP peaks with 50% reciprocal overlap within time points and between time points were considered true-positive regions. True-negative regions were derived from DNase-seq of HT29 cells (GSE90403)^55^. Regions with 50% reciprocal overlap between the two available DNase-seq biological replicates were identified, and all ChIP peaks from the corresponding NR ChIP datasets were then subtracted from the DNase-seq regions. Regions matched in size to each ChIP-derived true-positive region were randomly chosen from ChIP-subtracted DNase-seq regions to create the true negative regions. Background nucleotide frequencies for calculating PWMs from PFMs were taken from the nucleotide distribution of the DNase-seq regions with 50% reciprocal overlap between the two replicates. To score sequences, the following formalism was used:

$$p_{i,j} = \frac{{f_{i,j} + s \ast b_i}}{{\mathop {\sum }\nolimits_i f_{i,j} + s}}$$

Probability of an A,C,G or T (*i* = 0,1,2,3 respectively) occurring at position j of the sequence being evaluated.

*f*_*i,j*_: frequency defining the position frequency matrix

*b*_*i*_: nucleotide background frequencies: A: 0.24; T: 0.24; C: 0.26; G: 0.26

*s*: pseudo-count to deal with zeros (s = 0.001)

The PWM score is the sum over all base positions (j) of the corresponding *S*_*i,j*_ values for a particular sequence:

$$S_{i,j} = {\mathrm{log}}_2(\frac{{p_{i,j}}}{{b_i}})$$

Area under the ROC curve (AUC) values are reported to quantify the enrichment, and a Wilcox-Mann-Whitney (WMW) U test was applied to calculate the significance of each AUC value. AUC and WMW U test values were calculated in the R statistical package using the wilcox.test function. All manipulations of genomic regions (identification of overlapping regions, region subtractions, etc.) were performed with BEDTools 2.26.0^56^.

To examine the motif enrichment of currently available models, we performed the ROC analyses described above with publicly available PFMs. Each PFM was normalized such that the nucleotide frequencies at each position sum to 1. The following models were used: LXRα (MA0494.1^29^; HOCOMOCO f1^28^), PPARγ (PPARγ^30^; M00512, M00515, M00528^39^; MA0065.1, MA0065.2, MA0066.1^30^; HOCOMOCO f1, HOCOMOCO s1^28^).

To examine motif enrichment for putative ‘active’ sites near differentially expressed genes, RNA-seq data from HT29 cells^16^ were used to identify regions that are likely to be actively controlling transcription. We re-analyzed the published RNA-seq data using DESeq2^57^ to identify genes upregulated upon agonist treatment compared to vehicle only (DMSO). Transcripts with a fold-change greater than 2 and adjusted *p*-values less than 0.01 were considered upregulated. For PPARγ, transcripts upregulated after both 24 and 48 h of rosiglitazone treatment were considered for further analysis. For LXRα, transcripts upregulated after 48 h of GW3965 and T0901317 treatment were considered for further analysis. For each NR, ChIP regions with 50% reciprocal overlap between replicates and time points and within the indicated regions associated with upregulated genes were considered active true positives for enrichment analysis. Regions matched in size to each active region were randomly chosen from the true-negative regions described above to create the true negative regions. ROC analyses were performed as described above.

### DNA shape analysis

Binding to spacer-sequence variants of five DR3 and five DR4 seed sequences was analyzed (Supplementary Fig. 7). For each DR3 seed sequence, the PBM z-scores of the seed sequence and corresponding 9 SNV sequences (i.e., sequences with base variants at positions B1, B2, or B3) were analyzed to identify the two highest affinity and the two lowest affinity sites for each of the five seeds, resulting in a total of ten high and ten low-affinity spacer variants. The same procedure was performed for the DR4 sequences and the corresponding 12 SNVs at positions B1, B2, B3, and B4. For each of the 10 spacer variants, the following DNA shape parameters were calculated at each base position using the TFBSshape server^38^: major groove width (MWG), helix twist (HelT), propeller twist (ProT), and roll. The distribution of the DNA shape parameters associated with high and low-affinity sequences were compared at each base position using a two-tailed *t*-test.

### Reporting summary

Further information on research design is available in the Nature Research Reporting Summary linked to this article.

## Supplementary information

Supplementary Information

Peer Review

Reporting Summary

Description of Additional Supplementary Files

Supplementary Data 1

Supplementary Data 2

Supplementary Data 3

Supplementary Data 4

Supplementary Data 5

Source Data

## Data Availability

A reporting summary for this Article is available as a Supplementary Information file. PBM data generated for this study have been deposited in the NCBI GEO database with the accession code GSE124910. Replicate averaged and z-score normalized fluorescence values for PBM data generated for this study are provided as the file Supplementary Data 4. The source data underlying Fig. 1–7 and Supplementary Figs. 1–9 are provided as a Source Data file. Data from NCBI GEO datasets GSE77039 and GSE90403 were analyzed in this Article. All data are available from the corresponding author upon reasonable request.

## References

[1] de Aguiar Vallim TQ, Tarling EJ, Edwards PA (2013). Pleiotropic roles of bile acids in metabolism. Cell Metab..

[2] Evans RM, Mangelsdorf DJ (2014). Nuclear receptors, RXR, and the big bang. Cell.

[3] Kliewer SA, Lehmann JM, Willson TM (1999). Orphan nuclear receptors: shifting endocrinology into reverse. Science.

[4] Calkin AC, Tontonoz P (2012). Transcriptional integration of metabolism by the nuclear sterol-activated receptors LXR and FXR. Nat. Rev. Mol. Cell Biol..

[5] Potthoff MJ, Kliewer SA, Mangelsdorf DJ (2012). Endocrine fibroblast growth factors 15/19 and 21: from feast to famine. Genes Dev..

[6] Juge-Aubry C (1997). DNA binding properties of peroxisome proliferator-activated receptor subtypes on various natural peroxisome proliferator response elements. Importance of the 5’-flanking region. J. Biol. Chem..

[7] Claessens F, Gewirth DT (2004). DNA recognition by nuclear receptors. Essays Biochem..

[8] Cotnoir-White D, Laperrière D, Mader S (2011). Evolution of the repertoire of nuclear receptor binding sites in genomes. Mol. Cell. Endocrinol..

[9] Weikum Emily R., Liu Xu, Ortlund Eric A. (2018). The nuclear receptor superfamily: A structural perspective. Protein Science.

[10] Kurokawa R (1993). Differential orientations of the DNA-binding domain and carboxy-terminal dimerization interface regulate binding site selection by nuclear receptor heterodimers. Genes Dev..

[11] Mader S (1993). The patterns of binding of RAR, RXR and TR homo- and heterodimers to direct repeats are dictated by the binding specificites of the DNA binding domains. EMBO J..

[12] Perlmann T, Rangarajan PN, Umesono K, Evans RM (1993). Determinants for selective RAR and TR recognition of direct repeat HREs. Genes Dev..

[13] Boergesen M (2012). Genome-wide profiling of liver X receptor, retinoid X receptor, and peroxisome proliferator-activated receptor in mouse liver reveals extensive sharing of binding sites. Mol. Cell. Biol..

[14] Rastinejad F, Huang P, Chandra V, Khorasanizadeh S (2013). Understanding nuclear receptor form and function using structural biology. J. Mol. Endocrinol..

[15] Lefterova MI (2010). Cell-specific determinants of peroxisome proliferator-activated receptor gamma function in adipocytes and macrophages. Mol. Cell. Biol..

[16] Savic D (2016). Distinct gene regulatory programs define the inhibitory effects of liver X receptors and PPARG on cancer cell proliferation. Genome Med..

[17] Daniel B (2014). The active enhancer network operated by liganded RXR supports angiogenic activity in macrophages. Genes Dev..

[18] Soccio RE (2015). Genetic variation determines PPARγ function and anti-diabetic drug response in vivo. Cell.

[19] Zhan L (2014). Genome-wide binding and transcriptome analysis of human farnesoid X receptor in primary human hepatocytes. PLoS ONE.

[20] Chatagnon A (2015). RAR/RXR binding dynamics distinguish pluripotency from differentiation associated cis-regulatory elements. Nucleic Acids Res..

[21] Berger MF (2006). Compact, universal DNA microarrays to comprehensively determine transcription-factor binding site specificities. Nat. Biotechnol..

[22] Näär AM (1991). The orientation and spacing of core DNA-binding motifs dictate selective transcriptional responses to three nuclear receptors. Cell.

[23] Umesono K, Murakami KK, Thompson CC, Evans RM (1991). Direct repeats as selective response elements for the thyroid hormone, retinoic acid, and vitamin D3 receptors. Cell.

[24] Zechel C, Shen XQ, Chambon P, Gronemeyer H (1994). Dimerization interfaces formed between the DNA binding domains determine the cooperative binding of RXR/RAR and RXR/TR heterodimers to DR5 and DR4 elements. EMBO J..

[25] Miyamoto T (1997). Inhibition of peroxisome proliferator signaling pathways by thyroid hormone receptor. Competitive binding to the response element. J. Biol. Chem..

[26] Katz RW, Subauste JS, Koenig RJ (1995). The interplay of half-site sequence and spacing on the activity of direct repeat thyroid hormone response elements. J. Biol. Chem..

[27] Kurokawa R (1994). Regulation of retinoid signalling by receptor polarity and allosteric control of ligand binding. Nature.

[28] Kulakovskiy IV (2018). HOCOMOCO: towards a complete collection of transcription factor binding models for human and mouse via large-scale ChIP-Seq analysis. Nucleic Acids Res..

[29] Khan A (2018). JASPAR 2018: update of the open-access database of transcription factor binding profiles and its web framework. Nucleic Acids Res..

[30] Isakova A (2017). SMiLE-seq identifies binding motifs of single and dimeric transcription factors. Nat. Methods.

[31] Chandra V (2008). Structure of the intact PPAR-gamma-RXR- nuclear receptor complex on DNA. Nature.

[32] Temple KA (2005). An intact DNA-binding domain is not required for peroxisome proliferator-activated receptor gamma (PPARgamma) binding and activation on some PPAR response elements. J. Biol. Chem..

[33] Rastinejad F, Perlmann T, Evans RM, Sigler PB (1995). Structural determinants of nuclear receptor assembly on DNA direct repeats. Nature.

[34] Bolotin E (2010). Integrated approach for the identification of human hepatocyte nuclear factor 4alpha target genes using protein binding microarrays. Hepatology.

[35] Lou X (2014). Structure of the retinoid X receptor α-liver X receptor β (RXRα-LXRβ) heterodimer on DNA. Nat. Struct. Mol. Biol..

[36] Andrabi M (2017). Predicting conformational ensembles and genome-wide transcription factor binding sites from DNA sequences. Sci. Rep..

[37] Zhou T (2015). Quantitative modeling of transcription factor binding specificities using DNA shape. Proc. Natl Acad. Sci. USA.

[38] Yang L (2014). TFBSshape: a motif database for DNA shape features of transcription factor binding sites. Nucleic Acids Res..

[39] Matys V (2006). TRANSFAC and its module TRANSCompel: transcriptional gene regulation in eukaryotes. Nucleic Acids Res..

[40] Fang B, Mane-Padros D, Bolotin E, Jiang T, Sladek FM (2012). Identification of a binding motif specific to HNF4 by comparative analysis of multiple nuclear receptors. Nucleic Acids Res..

[41] Everett LJ, Lazar MA (2013). Cell-specific integration of nuclear receptor function at the genome. Wiley Inter. Rev. Syst. Biol. Med.

[42] Meijsing SH (2009). DNA binding site sequence directs glucocorticoid receptor structure and activity. Science.

[43] Schöne S (2016). Sequences flanking the core-binding site modulate glucocorticoid receptor structure and activity. Nat. Commun..

[44] Gronemeyer H, Bourguet W (2009). Allosteric effects govern nuclear receptor action: DNA appears as a player. Sci. Signal..

[45] Hall JM, McDonnell DP, Korach KS (2002). Allosteric regulation of estrogen receptor structure, function, and coactivator recruitment by different estrogen response elements. Mol. Endocrinol..

[46] Watson LC (2013). The glucocorticoid receptor dimer interface allosterically transmits sequence-specific DNA signals. Nat. Struct. Mol. Biol..

[47] Velasco LFR (2007). Thyroid hormone response element organization dictates the composition of active receptor. J. Biol. Chem..

[48] Hudson WH (2018). Cryptic glucocorticoid receptor-binding sites pervade genomic NF-κB response elements. Nat. Commun..

[49] Issa LL, Leong GM, Barry JB, Sutherland RL, Eisman JA (2001). Glucocorticoid receptor-interacting protein-1 and receptor-associated coactivator-3 differentially interact with the vitamin D receptor (VDR) and regulate VDR-retinoid X receptor transcriptional cross-talk. Endocrinology.

[50] Lefebvre P, Mouchon A, Lefebvre B, Formstecher P (1998). Binding of retinoic acid receptor heterodimers to DNA. A role for histones NH2 termini. J. Biol. Chem..

[51] Berger MF, Bulyk ML (2009). Universal protein-binding microarrays for the comprehensive characterization of the DNA-binding specificities of transcription factors. Nat. Protoc..

[52] Andrilenas KK (2018). DNA-binding landscape of IRF3, IRF5 and IRF7 dimers: implications for dimer-specific gene regulation. Nucleic Acids Res..

[53] Kuzmic P (1996). Program DYNAFIT for the analysis of enzyme kinetic data: application to HIV proteinase. Anal. Biochem..

[54] Golden MS (2013). Comprehensive experimental and computational analysis of binding energy hot spots at the NF-κB essential modulator/IKKβ protein-protein interface. J. Am. Chem. Soc..

[55] Consortium TEP (2013). An integrated encyclopedia of DNA elements in the human genome. Nature.

[56] Quinlan AR, Hall IM (2010). BEDTools: a flexible suite of utilities for comparing genomic features. Bioinformatics.

[57] Love MI, Huber W, Anders S (2014). Moderated estimation of fold change and dispersion for RNA-seq data with DESeq2. Genome Biol..

