# Supplementary material for: Comprehensive study of nuclear receptor DNA binding provides a revised framework for understanding receptor specificity
Source: Nat Commun. 2019 Jun 7;10:2514. doi: 10.1038/s41467-019-10264-3 (PMC6555819; doi:10.1038/s41467-019-10264-3)
Supplement: Supplementary file 1 — Supplementary Information [file 41467_2019_10264_MOESM1_ESM.pdf]

Supplementary Information for

Comprehensive Study of Nuclear Receptor DNA Binding Provides a Revised Framework for Understanding Receptor Specificity

Penvose and Keenan et al.

Supplementary figure 1

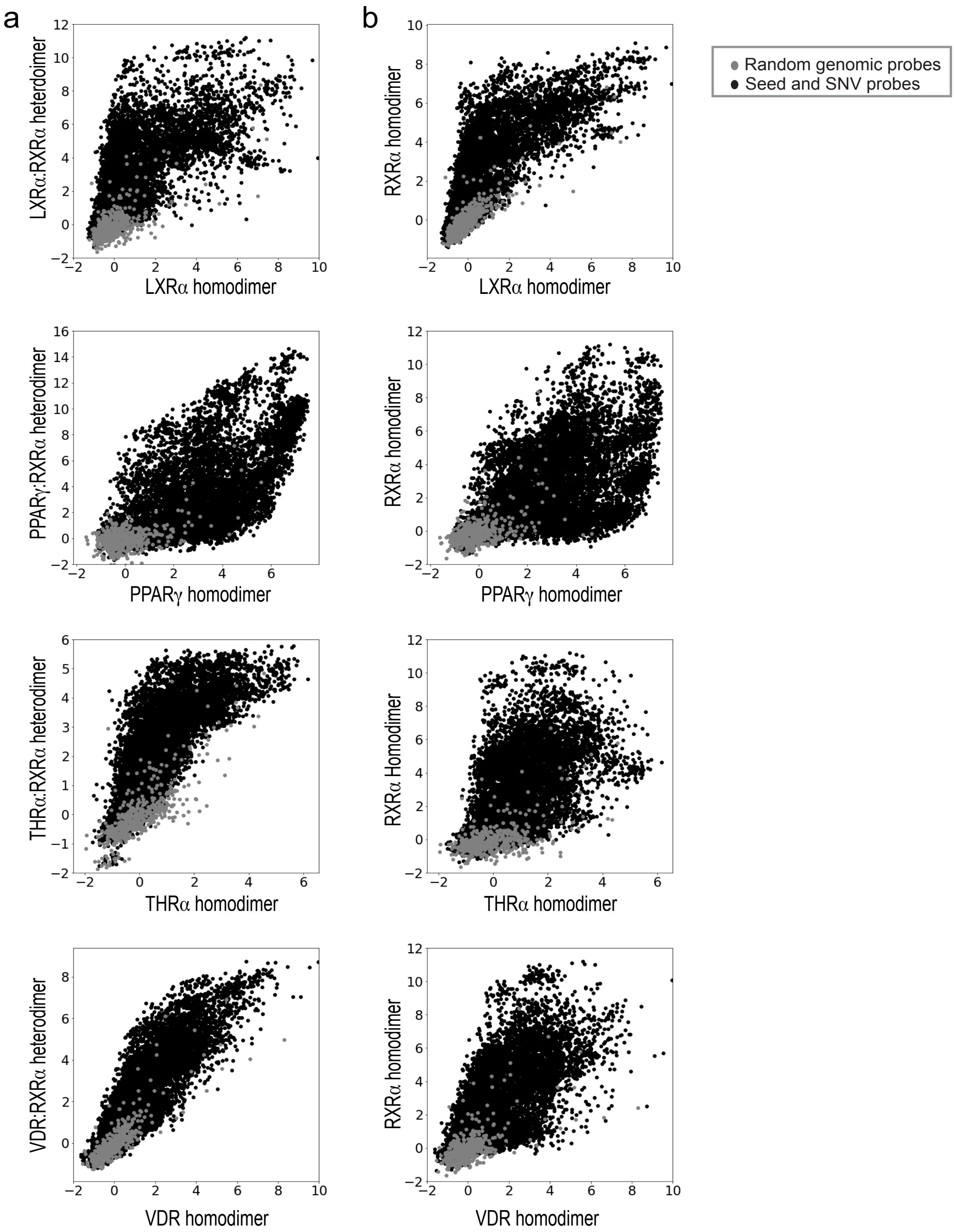

Supplementary figure 1. Comparison of NR homodimer and heterodimer binding. Z-scores for **a** NR as a heterodimer with RXR against the corresponding NR homodimers or **b** for RXR homodimer against NR homodimer. Dots represent average over ~5 replicates for all 10,728 unique SNV probes and 500 background probes. Source data are provided as a Source Data file.

Supplementary figure 2

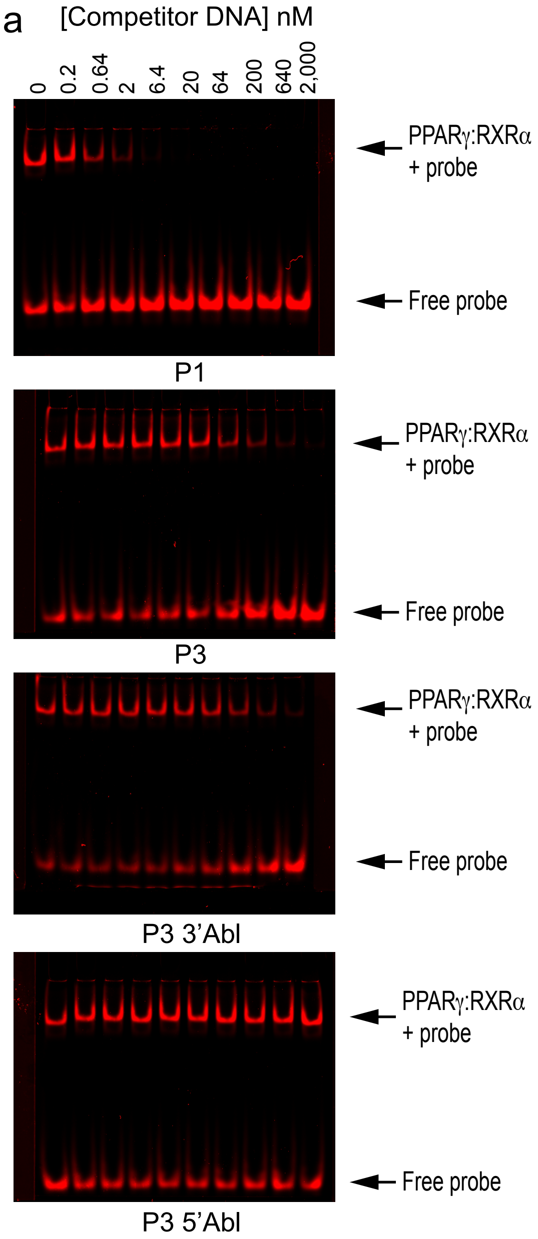

b

Competitor DNA sequences:

P1: ACT**AGGTCAAAGGTCA**AAG

P3: ACA**GGGTCAAAGGCCA**GAG

P3 5'Abl: ACACCGTCAA**AGGCCA**GAG

P3 3'Abl: ACA**GGGTCA**AAGGCGAGAG

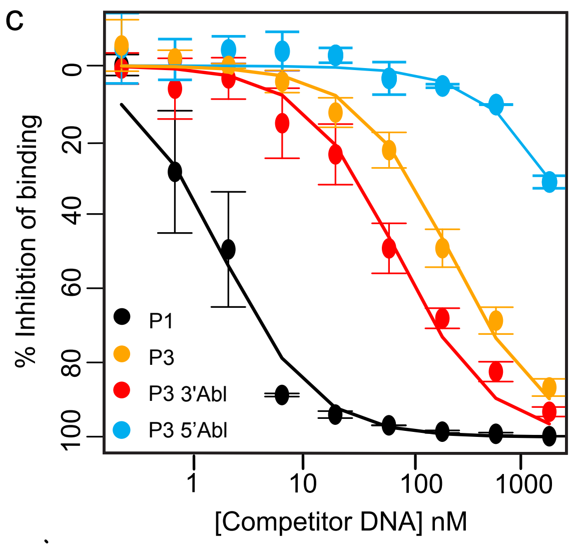

Supplementary figure 2. Competion EMSA experiments for PPARγ:RXRα **a** Representative EMSA gels of competition for binding by PPARγ:RXRα to labeled DNA probe (P1, as described in Fig. 1g) and four unlabeled competitor DNA sequences whose sequence are shown in **b**. **c** Inhibition curves determined by quantifying the intensity of the bound probe band at different competitor concentrations for the different competitor experiments (error=STDEV, n = 2). Source data are provided as a Source Data file.

Supplementary figure 3

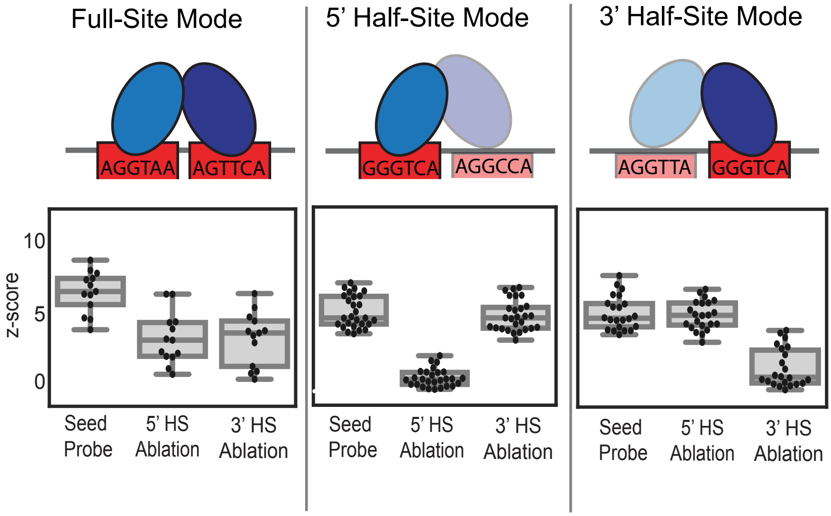

Supplementary figure 3. Impact of half-site ablation on LXRα binding. Z-score distribution of LXRα binding to seed probes bound in the full-site mode or half-site mode, and the z-score distributions for binding to corresponding sequences with 5' or 3' half-site ablations. Source data are provided as a Source Data file.

Supplementary figure 4

a

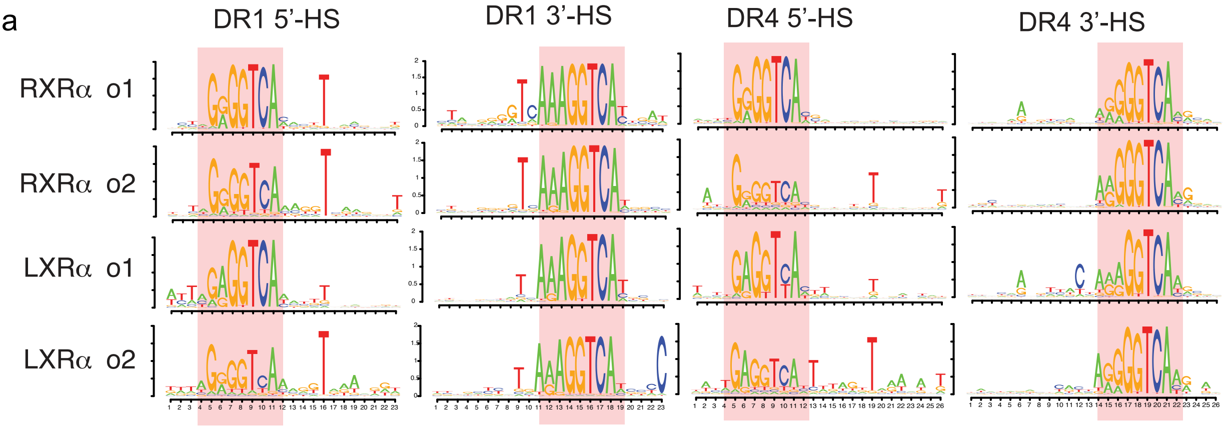

b

Seed sequence orientations relative to glass slide

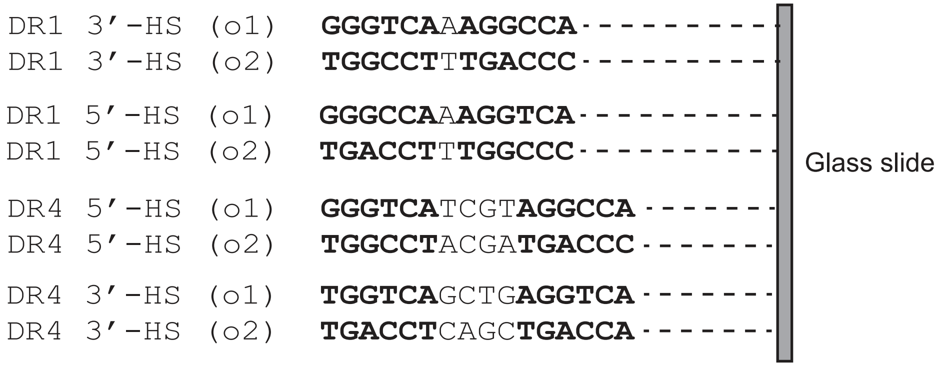

Supplementary figure 4. Impact of PBM probe orientation on NR binding logos. **a** DNA binding logos for RXRα homodimers and LXRα:RXRα are shown for DNA sequences bound in either a 5' half-site or 3' half-site binding mode. DNA binding logos were determined separately from PBM probes in which the binding site (and all SNVs used in the logo determination) are oriented in either the o1 or o2 orientation with respect to the glass slide (schematized in **b**). Bases indicating the binding mode preference are highlighted with the red overlay box. **b** Schematic of DNA seed sequences used to generate the logos showing the orientation relative to the microarray glass slide. Source data are provided as a Source Data file.

Supplementary figure 5

a

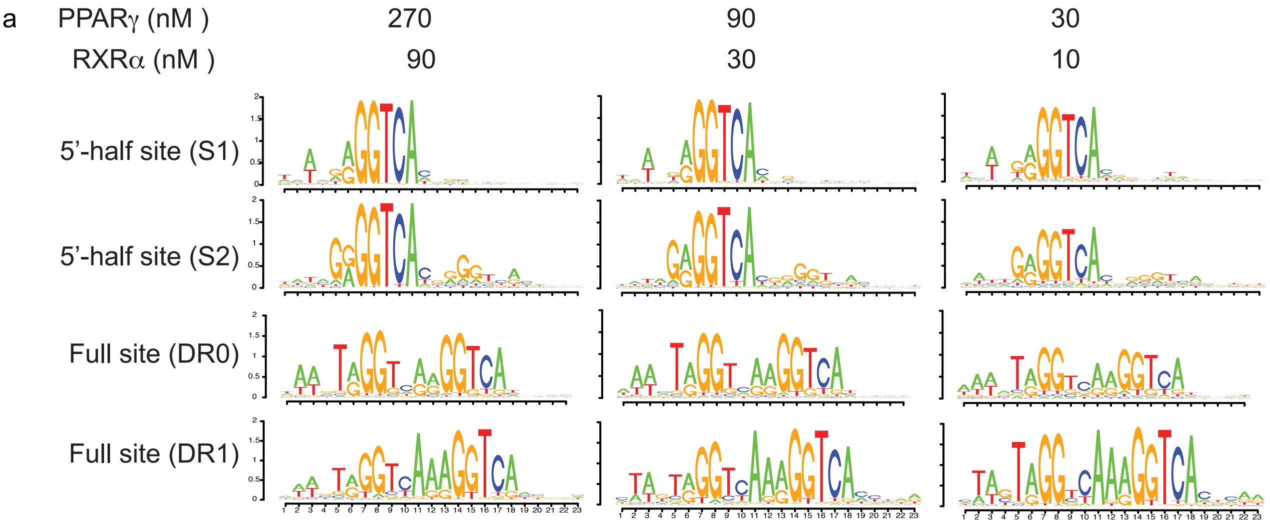

b Seed Sequences for Motif Models

5'-half site (S1)	GAACT**AGGTCA**CC**AGGACA**GTGAA
5'-half site (S2)	ATACA**GGGTCA**CT**AGGCCA**GAGTA
Full site (DR0)	TATGT**AGGTTAGGGTCA**TTCAG
Full site (DR1)	CTAGC**AGGCCA**A**GGGTCA**CTCAA

Supplementary figure 5. Impact of protein concentration on NR binding logos. **a** PPARγ:RXRα DNA binding logos for DNA seed sequences bound in full or half-site binding modes are shown for PBM experiments performed at three different concentrations. The concentration of each monomer used in each PBM experiment is indicated. **b** The seed sequences for which the logos in **a** were generated. Identifiable DR half-sites in each binding sequence are shown in bold. Source data are provided as a Source Data file.

Supplementary figure 6

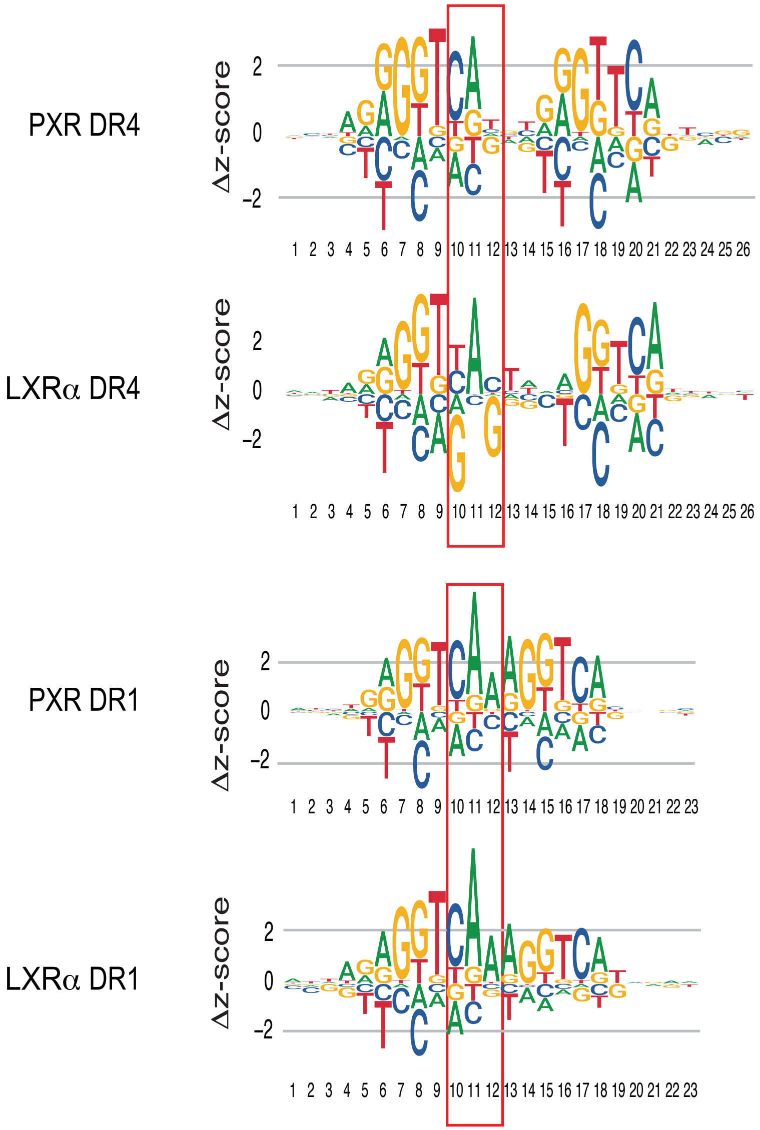

Supplementary figure 6. DNA energy matrix logos for LXRα and PXR. DR1 and DR4 logos, directly representing Δz-scores of SNV binding, are shown for LXRα and PXR. DR4 logos are derived from the same experiments as those in Fig. 5 and are shown for comparison. Positive Δ z-scores indicate z-scores higher than the median z-score for all base variants at that position. Source data are provided as a Source Data file.

Supplementary figure 7

a

DR3 seed sequences

B1 B2 B3

AGGTCA**CAG**AGGTCA
AGGTCA**GAG**AGGTCA
TGGTCA**CGG**AGGTCA
AGTTCA**TCT**AGGTAA
GGGTCA**GCT**AGGCCA

DR4 seed sequences

B1 B2 B3 B4

AGGTCA**CGAA**AGGTCA
AGGTCA**TAGG**AGGTCA
TGGTCA**GCTG**AGGTCA
AGTTCA**ACTA**AGGTAA
GGGTCA**TCGT**AGGCCA

b

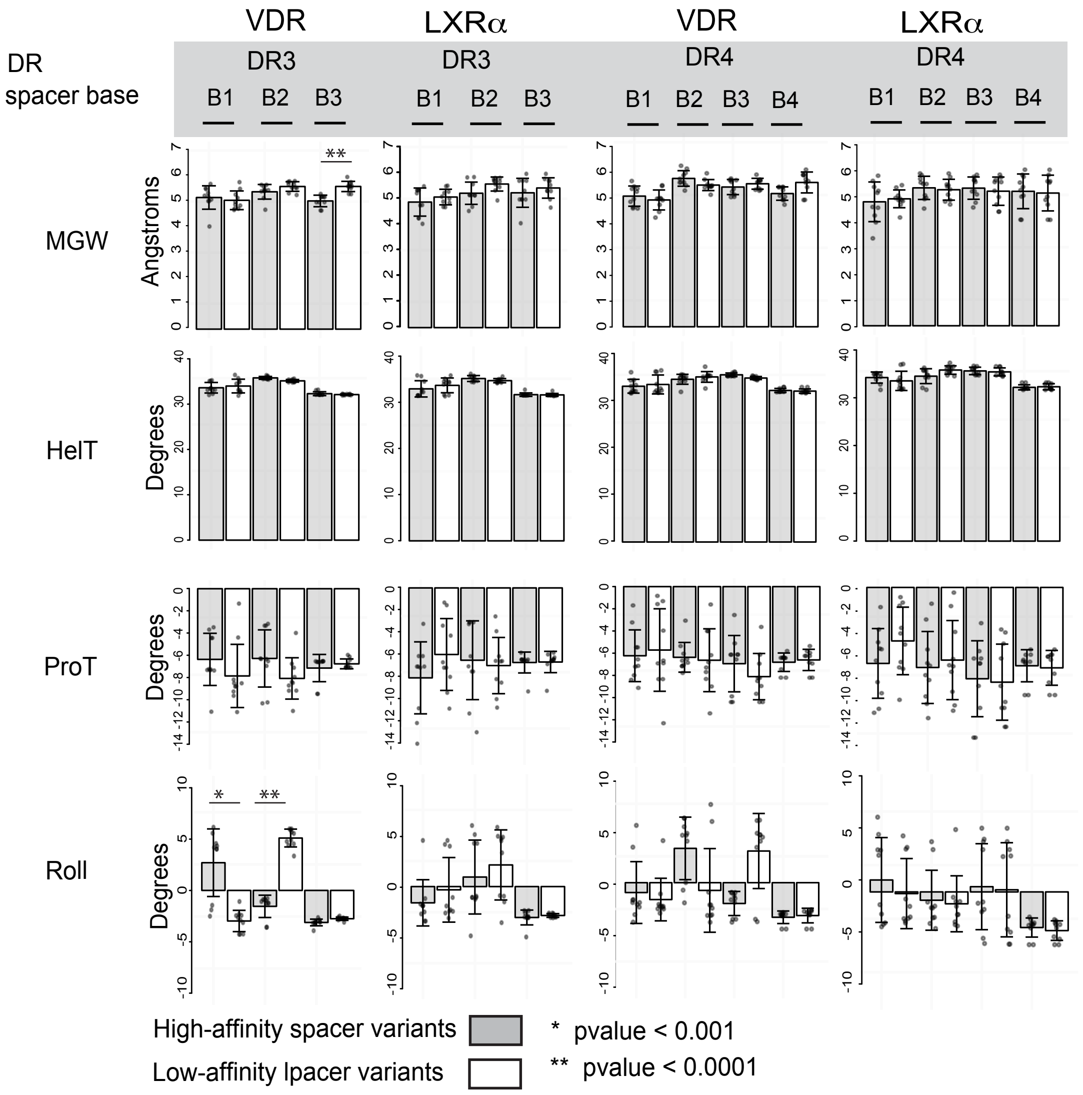

Supplementary figure 7. DNA-shape parameters of spacer sequences for high and low-affinity NR binding sites. **a** Schematic of DNA seed sequences used to analyze DNA shape features (shown in b). Base positions in the spacer sequence between the DR half-sites are indicated in bold and referred to as B1,B2,B3 (DR3 site) and B1,B2,B3,B4 (DR4 site). Seed sequences were selected to represent diverse spacer sequences. **b** Distribution of DNA shape features for spacer sequences in either high-affinity sites (grey bars) or low-affinity sites (white bars). Data is shown for VDR and LXRα heterodimer binding experiments. For each of the 5 seed sequences (at each spacer length), we identified the two highest affinity and the two lowest affinity spacer sequence variants. Therefore, there are 10 (i.e, 5x2) high-affinity and 10 low-affinity spacer sequences considered for each bar plot. For each of the 10 spacer variants, DNA shape parameters were calculated at each base position using the TFBSshape server[38] – major groove width (MWG), helix twist (HelT), propeller twist (ProT), and roll. Shown at each base position is the mean parameter over 10 sequences (error = STDEV). Distributions that were significantly different between the high and low-affinity sequences are shown (p-value calculated using a two-tailed t-test). Source data are provided as a Source Data file.

Supplementary figure 8

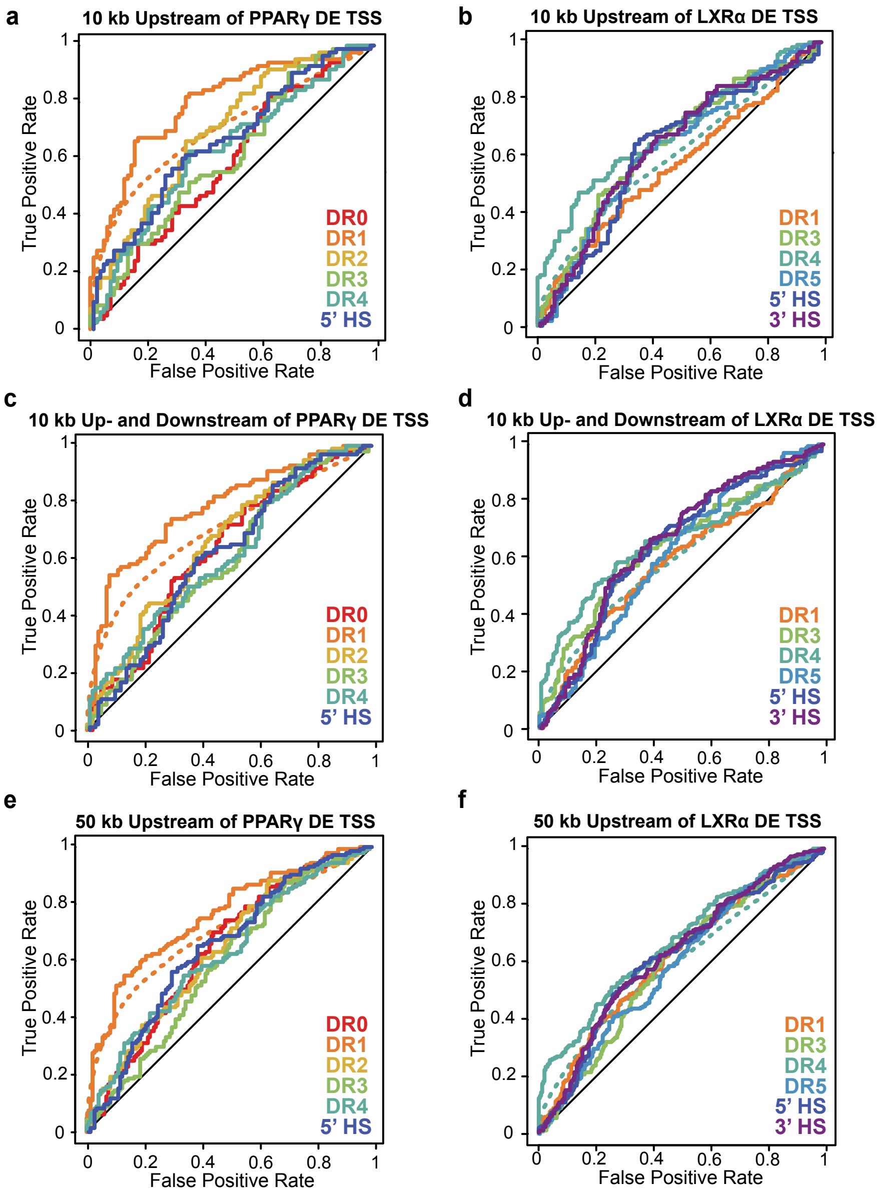

Supplementary figure 8. Receiver operating characteristic (ROC) curves for PPARγ and LXRα motif enrichment in ChIP-seq data. ROC curves for motif enrichment of PBM-derived PPARγ-binding models are shown for all reproducibly-bound PPARγ ChIP-seq peaks (dotted lines, **a,c,e**) and reproducibly-bound PPARγ ChIP-seq peaks occurring within **a** 10 kb upstream, **c** 10 kb upstream or downstream, and **e** 50 kb upstream of the transcription start site of differentially expressed genes (solid lines, Methods). ROC curves for motif enrichment of PBM-derived LXRα binding models are shown for all reproducibly-bound LXRα ChIP-seq peaks (dotted lines, **b,d,e**) and reproducibly-bound LXRα ChIP-seq peaks occurring within **b** 10 kb upstream, **d** 10 kb upstream or downstream, **f** and 50 kb upstream of the transcription start site of differentially expressed genes (solid lines, Methods). ROC curves determined using different PWMs for different DR and half-site (HS) modes are indicated. Source data are provided as a Source Data file.

Supplementary figure 9

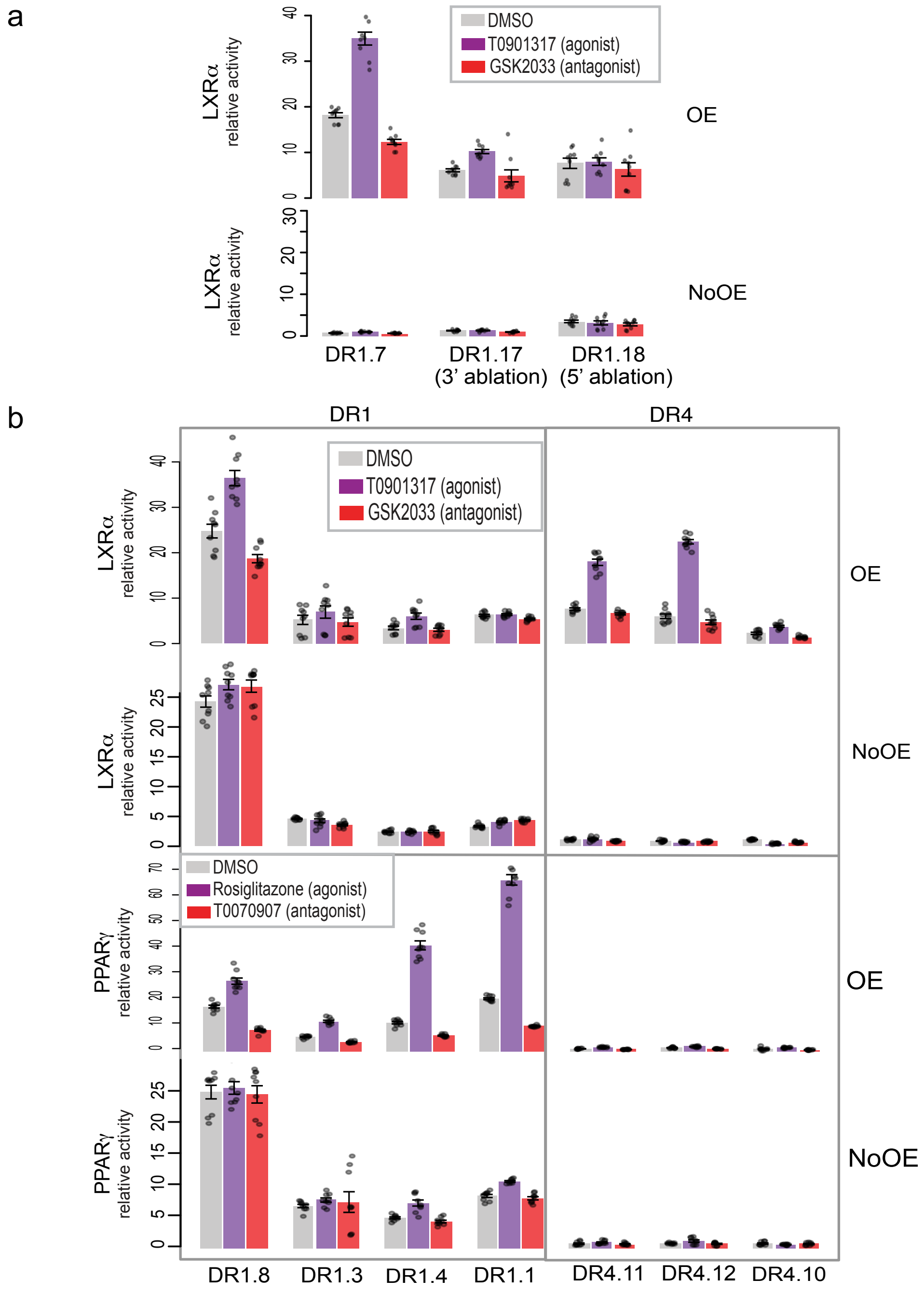

Supplementary figure 9. Impact of NR over-expression on reporter gene activity. **a,b** LXRα- and PPARγ-dependent activity for the sequences described in Fig. 7 in the same treatment conditions. Shown separately are the luciferase activity values for the cells in which the NR:RXRα proteins were overexpressed (OE) and the values in which the proteins were not overexpressed (NoOE), each normalized to empty vector. Fold-change values in Fig. 7 are the ratio of these sets of values (i.e., OE/NoOE). Values represent the mean over nine replicate measurements (error bars = SEM). Source data are provided as a Source Data file.
