# Supplementary material for: Comprehensive study of nuclear receptor DNA binding provides a revised framework for understanding receptor specificity
Source: Nat Commun. 2019 Jun 7;10:2514. doi: 10.1038/s41467-019-10264-3 (PMC6555819; doi:10.1038/s41467-019-10264-3)
Supplement: Supplementary file 3 — Reporting Summary [file 41467_2019_10264_MOESM3_ESM.pdf]

nature research

Corresponding author(s): *Double-blind peer review submissions: write DBPR and your manuscript number here instead of author names.*

Reporting Summary

Nature Research wishes to improve the reproducibility of the work that we publish. This form provides structure for consistency and transparency in reporting. For further information on Nature Research policies, see Authors & Referees and the Editorial Policy Checklist.

Statistical parameters

When statistical analyses are reported, confirm that the following items are present in the relevant location (e.g. figure legend, table legend, main text, or Methods section).

n/a	Confirmed	
☐	☒	The exact sample size (n) for each experimental group/condition, given as a discrete number and unit of measurement
☐	☒	An indication of whether measurements were taken from distinct samples or whether the same sample was measured repeatedly
☐	☒	The statistical test(s) used AND whether they are one- or two-sided<br>*Only common tests should be described solely by name; describe more complex techniques in the Methods section.*
☒	☐	A description of all covariates tested
☐	☒	A description of any assumptions or corrections, such as tests of normality and adjustment for multiple comparisons
☐	☒	A full description of the statistics including central tendency (e.g. means) or other basic estimates (e.g. regression coefficient) AND variation (e.g. standard deviation) or associated estimates of uncertainty (e.g. confidence intervals)
☒	☐	For null hypothesis testing, the test statistic (e.g. F, t, r) with confidence intervals, effect sizes, degrees of freedom and P value noted<br>*Give P values as exact values whenever suitable.*
☒	☐	For Bayesian analysis, information on the choice of priors and Markov chain Monte Carlo settings
☒	☐	For hierarchical and complex designs, identification of the appropriate level for tests and full reporting of outcomes
☐	☒	Estimates of effect sizes (e.g. Cohen's d, Pearson's r), indicating how they were calculated
☐	☒	Clearly defined error bars<br>*State explicitly what error bars represent (e.g. SD, SE, CI)*

Our web collection on statistics for biologists may be useful.

Software and code

Policy information about availability of computer code

Data collection	-Microarray fluorescence data was collected using GenePix Pro 7.2.<br>-Fluorescence EMSA data was acquired and quantified with ImageStudioLite software.<br>-Luciferase reporter data was quantified using a VICTOR-3 plate reader and accompanying VICTOR-3 software.

Data analysis

PBM Data:
-Microarray fluorescence data was normalized using MicroArray LINEar Regression (doi: 10.1385/1-59745-097-9:245).
-Microarray fluorescence data was spatially de-trended as described previously (doi: 10.1038/nprot.2008.195).
-DNA shape features were calculated with TFBSshape (doi: 10.1093/nar/gkt1087).

ChIP-seq and RNA-seq Data:
-DESeq2 (doi: 10.1186/s13059-014-0550-8) was used for differential expression analysis.
-BEDTools 2.26.0 (doi: 10.1093/bioinformatics/btq033) was used for all manipulations of genomic regions (identification of overlapping regions, region subtractions, etc.)
-Custom perl and R scripts were used to perform ROC analyses. These scripts will be made publicly available upon request.

EMSA Data:
-Kd values were calculated with DynaFit 4 software.

For manuscripts utilizing custom algorithms or software that are central to the research but not yet described in published literature, software must be made available to editors/reviewers upon request. We strongly encourage code deposition in a community repository (e.g. GitHub). See the Nature Research guidelines for submitting code & software for further information.

Data

Policy information about availability of data

All manuscripts must include a data availability statement. This statement should provide the following information, where applicable:

- Accession codes, unique identifiers, or web links for publicly available datasets
- A list of figures that have associated raw data
- A description of any restrictions on data availability

All relevant data are publicly available. PBM data generated for this study have been deposited in the NCBI GEO database with the accession code GSE124910 (https://www.ncbi.nlm.nih.gov/geo/query/acc.cgi?acc=GSE124910). Replicate averaged and z-score normalized fluorescence values for PBM data generated for this study are provided as the file Supplementary Data 4. The source data underlying Figures 1-7 and Supplementary Figures 1-9 are provided as a Source Data file. Data from NCBI GEO datasets GSE77039 (https://www.ncbi.nlm.nih.gov/geo/query/acc.cgi?acc=GSE77039) and GSE90403 (https://www.ncbi.nlm.nih.gov/geo/query/acc.cgi?acc=GSE90403) were analyzed in this Article. A reporting summary for this Article is available as a Supplementary Information file.

Field-specific reporting

Please select the best fit for your research. If you are not sure, read the appropriate sections before making your selection.

☒ Life sciences ☐ Behavioural & social sciences ☐ Ecological, evolutionary & environmental sciences

For a reference copy of the document with all sections, see nature.com/authors/policies/ReportingSummary-flat.pdf

Life sciences study design

All studies must disclose on these points even when the disclosure is negative.

Sample size

Reporter assays were performed as three biological replicates with three technical replicates per biological replicate for each sequence tested.

For ROC analyses, sample size was based on the number of reproducible peaks from the ChIP-seq data utilized. The methods used to classify peaks as reproducible are detailed fully in the methods section.

All protein-binding microarray experiments were done in replicate on separate microarrays. Each single nucleotide variant sequence is included as five replicates on each chamber of the array and each genomic background sequence is included as four replicates on each chamber of the array.

Data exclusions

No data was excluded.

Replication

For reporter assays, three biological replicates and three technical replicates were performed for each sequence tested. The mean and standard error of the mean for each test sequence are reported. The results were highly reproducible and no replicates were excluded.

For heterodimer PBM experiments, each protein was tested in replicate, with one replicate probed with an antibody recognizing RXR and the other replicate probed for the partner nuclear receptor using an anti-His antibody. The R^2 between replicates was checked to measure reproducibility, and those values are provided in Supplementary Data 1. GSE124910 includes the independent measurements with each antibody, and the replicate averaged data. Replicate averaged data for the PBM is also provided as Supplementary Data 4. All PBM experiments were highly reproducible.

Randomization

No randomization was used.

Blinding

No blinding was used.

Reporting for specific materials, systems and methods

Materials & experimental systems

n/a	Involved in the study
	☒ Unique biological materials
	☒ Antibodies
	☒ Eukaryotic cell lines
☒	☐ Palaeontology
☒	☐ Animals and other organisms
☒	☐ Human research participants

Methods

n/a	Involved in the study
☒	☐ ChIP-seq
☒	☐ Flow cytometry
☒	☐ MRI-based neuroimaging

Unique biological materials

Policy information about availability of materials

Obtaining unique materials

All unique materials produced in our lab, including protein expression vectors, are readily available from the author.
Proteins obtained from commercial sources are listed below and are listed in Supplementary Data 1.

Vendor source and product number for commercially available proteins:
FXR (Protein One P1044-01), LXRα (Protein One P1045-01), LXRβ (Abcam ab81924), PPARα (Abcam ab81925), PPARγ (Protein One P1050-01), PXR (Protein One R1082-02), RARα (Protein One P1054-01), RARβ (Protein One P1056-01), RARγ (Protein One P1055-02), THRα (Protein One P1052-02), and VDR (Protein One R1084-2). This info is also detailed in Supplementary Data 1.

Antibodies

Antibodies used

Anti-RXRα antibody (Active Motif 61029, Clone: 4RX-3A2, Lot#33616002)
Anti-His antibody (Sigma H1029, Clone: His-1, Lot#075M4847V)
Anti-PPARγ antibody (Abcam 41928, Lot # GR26030-53)
Alexa488-conjugated anti-GST antibody (Life Tech A11131)

Validation

Anti-RXRα antibody: Reactivity: Human, validated for EMSA (a methodology similar to PBM in that it recognizes native protein bound to DNA)
Anti-His antibody: As this is a synthetic epitope, species reactivity does not apply; antibody recognizes synthetic polyhistidine, as well as native or denatured, reduced forms of proteins tagged with 6X histidines
Anti-PPARγ antibody: Reactivity: human, mouse, rat; validated for ChIP and EMSA (a methodology similar to PBM in that it recognizes native protein bound to DNA)
Anti-GST antibody: As this is standard tag, species reactivity does not apply; antibody recognizes GST tag

Eukaryotic cell lines

Policy information about cell lines

Cell line source(s)	293T/17 [HEK 293T/17] (ATCC® CRL-11268) were obtained directly from ATCC
Authentication	No authentication was performed by us as fresh cells were obtained directly from ATCC
Mycoplasma contamination	Cell line was not tested for mycoplasma contamination.
Commonly misidentified lines (See ICLAC register)	*Name any commonly misidentified cell lines used in the study and provide a rationale for their use.*
