# Supplementary material for: Comprehensive study of nuclear receptor DNA binding provides a revised framework for understanding receptor specificity
Source: Nat Commun. 2019 Jun 7;10:2514. doi: 10.1038/s41467-019-10264-3 (PMC6555819; doi:10.1038/s41467-019-10264-3)
Supplement: Supplementary file 4 — Description of Additional Supplementary Files [file 41467_2019_10264_MOESM4_ESM.docx]

**Description of Additional Supplementary Files**

File Name: Supplementary Data 1
Description: Sources and concentrations of proteins used for all PBM experiments

File Name: Supplementary Data 2
Description: Literature curated response elements (Figures 1b&e), EMSA probe sequences (Fig 1g and S1), and reporter sequences (Figures 7a-c)

File Name: Supplementary Data 3
Description: Published PWM models used in Figure 1b

File Name: Supplementary Data 4.
Description: Replicate averaged and z-score normalized fluorescence values for PBM data

File Name: Supplementary Data 5
Description: Curation of binding models for wild-type and DNA-binding domain (DBD) mutant PBM experiments
