# Supplementary material for: Comprehensive study of nuclear receptor DNA binding provides a revised framework for understanding receptor specificity
Source: Nat Commun. 2019 Jun 7;10:2514. doi: 10.1038/s41467-019-10264-3 (PMC6555819; doi:10.1038/s41467-019-10264-3)
Supplement: Supplementary file 2 — Peer Review [file 41467_2019_10264_MOESM2_ESM.pdf]

Reviewers' comments:

Reviewer #1 (Remarks to the Author):

Penvose, et al. examine the sequence specificity of Type II nuclear receptors (NR) that bind DNA as heterodimers with a common partner (RXRa). While confirming the preferences for known direct repeat(DR) binding site arrangements, they also report unexpected promiscuity in binding of all 11 heterodimers and the RXRa homodimer to the wider set of DRs (repeats spaced by 0-5 intervening bases). By motif building, site ablation and protein mutation they demonstrate that heterodimers likely bind to different DR arrangements (DR 0-5) by associating with a single repeat or a half site. The two modes of interaction neatly explain the genome-wide ChIP-seq studies which often find that a small fraction of the ChIP peaks contain an ideal DR repeat and much larger fraction contains half sites. The authors go on examine the role of half site binding in ligand-responsive reporter gene expression. Their results confirm that half site binding may permit inducible gene expression but optimal DR sites may result in higher ligand-responsive expression of the reporter. Their data lead them to draw a distinction between high affinity sites and high activity sites, emphasizing that high affinity in vitro or in vivo does not predict higher gene-induction in cells.

Taken together this study provides an important step forward in understanding the function of type II NRs in cells, therefore it certainly merits publication. However, some of the results do raise concerns that should be addressed to ensure that interpretations are made on data that are not influenced by the experimental platform or computational models that were utilized in this study.

An important technical concern is that promiscuity in binding to different DR sites may be a consequence of performing the binding experiment on the protein-binding microarray (PBM) platform. For example, the density of DNA probes on the surface and low rate of mass transport at surfaces generate non-equilibrium conditions and may enhance NR binding to sites that may not otherwise be bound in solution. Moreover, low level aggregation of recombinant NRs (given the 3:1 ratio of the two partners) may enable multi-site binding across different DNA probes reducing the discrimination between different DRs. To address these type of platform-based concerns, it would be necessary to demonstrate binding to the range of binding sites using other methods such as electrophoretic mobility shift assays or solution-based fluorescence polarization assays. Different concentrations of the protein complex should be tested to determine if their PBM-based DR promiscuity diminishes at lower protein concentrations or more stringent in vitro binding conditions.

While the authors examine binding of each partner to the set of PBM probes and report high correlation, suggestive of heterodimer binding, it is important to demonstrate that heterodimers are forming and to isolate heterodimers by standard chromatographic processes and validate the binding profiles for at least a handful of proteins that have been reported to binding DNA as monomers. This is particularly important because of the high level of half site binding that they observe. Both NRs and RXRa can bind similar half sites with similar affinities.

The most striking conclusion of their study is that nearly all NRs bind half sites and this leads to high affinity associations with different DRs. To emphasize their point, the authors display one subunit of the dimer not making contact with DNA. This may be misleading as the very first crystal structure of GR homodimer by Paul Sigler revealed one monomer making base-specific contacts while the other monomer docked on to DNA without making such contacts but retaining electrostatic interactions with the DNA backbone. More important, it is unclear why 5' half sites are different than 3' half sites. Given that the sites are direct repeats and based on their probe design, the dimer should be able slide/hop over to the next repeat (5' or 3') and associate indistinguishably with the duplex. The exception to this possibility is the 5' half site preference displayed by PPARg. The authors do not adequately address this worrying anomaly. This result raises the concern that arrangement of the binding sites with respect to the glass side may influence binding to the more solvent-accessible 5' half site. The extent to which the PBM platform influences binding of NR heterodimers has not been rigorously evaluated.

Previous reports (Weirauch et al Nature Biotech 2014) had reported that proximity to the slide had an impact on accessibility and could skew binding preferences. It is imperative that such half site preferences are examined by alternative methods, such as MITOMI, that eliminate any potential for biased protein associations on surface-immobilized short DNA duplexes.

In an elegant series of experiments in Figure 3, the authors try to parse out which partner enables half site binding. The data in Figure 3C is clear but 3D seems redundant and best moved to supplemental materials. (On a minor note, the supplementary figures and data are poorly described and seem to have suffered some form or formatting glitch. It would be helpful to the audience to have this material more accessible.)

The RXRa mutation demonstrates half site binding by PPARg but it also increases the preference for TGT upstream of the half site (Fig 3G). This preference is different than in Fig 3E. Why would such a preference for 5' flanks appear when RXRa is mutated? The reciprocal mutations in PPARg and LXRa would help clarify if this is a latent preference of those particular NRs or if this is a general feature of NR heterodimers. I would highly recommend this experiment as it would also address why nearly 50% of the sites that are bound in the half-site mode permit binding by PPARg whereas the other half are only bound by RXRa. Such clear difference in the ability each partner to associate with specific half sites is extremely odd and needs to be addressed by the authors.

If differences between half sites are not apparent from their own computational method, the authors may consider methods such as No-reads left behind (Bussemaker) to see if other NR-specific specificity determinants are identified that explain the differential association of each partner with different half sites. NRLB may eliminate the need for an arbitrary z-score cut-off (>3) and reveal specificity determinants that might be more evident in lower affinity sites.

Given the recent emphasis on the importance of DNA shape, it is unclear why the authors did not explore the potential role for shape in explaining the different preference of VDR for different DR3 sites or LXRa for different DR4 sites. It would enrich their study to include this analysis of the spacer. Similar analysis of Hox-Exd heterodimers by the Bussemaker and Mann labs revealed latent preferences of different Hox factors.

Finally, there are a few minor issues that should be addressed. These include, reporting excessive p-values as in Fig. 6A, overstating the differences between z-scores as in the case of Fig. 7C, and arbitrary definition of "functional sites" (searching for motifs 10Kb upstream of start sites and ignoring intronic enhancers or motifs that lie beyond 10kb).

Reviewer #2 (Remarks to the Author):

The authors use the powerful protein binding microarray (PBM) technology to examine the DNA binding specificity of 12 nuclear receptors that bind DNA as heterodimeric partners with RXR. More than 25 years ago the DR (direct repeat) rule for NR DNA binding was formulated based on a handful of DNA binding sites that had been identified for various NRs at the time. It was an incredibly insightful breakthrough that allowed the field to deal with the complexity of 100s of NRs found in every animal organism and their 10,000s of target genes. However, this elegant rule did not address the biological reality that several NRs bound the same type of DR motifs (e.g., DR1, DR2, DR3 etc) and yet had very different functions in vivo, nor the fact that calling a given site a DR1,2,3, etc versus a variation on a single DR type motif was often in the mind of the beholder. Furthermore, nearly all the classical in vitro studies were dependent on gel shift assays; polyacrylamide gels and substantial electrical fields are not found in the nucleus. The advent of in vivo genome-wide DNA binding methods such as ChIPseq did not necessarily clarify things as predicting exactly which DNA sequence of 12-15 nucleotides is responsible for the binding of a NR to region of DNA 100 or more nucleotides is not an

exact science, even though there are plenty of algorithms that predict binding motifs.

In this work the authors correlate in vitro PBM DNA binding data to 10,000s of sequences with luciferase assays as well as in vivo ChIPseq and RNAseq data to dig deeper into the DR rule for NRs. Whereas they find compelling evidence for the DR rules, they also find exceptions as well as binding to what appears to be half sites, although a cursory analysis suggests that the canonical sites based on the DR rules are the most active in vivo. Overall the manuscript is very well written and easy to follow; however, there are several major as well as minor issues that need to be addressed.

Major issues

1. The conclusions from Fig. 7 and the presentation of the model in Fig. 7D are not consistent with their data, and the text is often confusing. The luciferase data in Fig. 7AC show that even though certain half sites yield good binding in vitro in the PBMs they do not result in good activation of transcription in vivo in luciferase assays. This is consistent with the data presented in Fig. 6CD (although it is not clear that half versus full-sites have been distinguished in these data). In contrast, in the model in Fig. 7D, they show the same level of activation by half sites as by full sites. There are also several contradictory statements in lines 396 thru 402 and lines 449-450 do not make sense. Lines 415-416 – this is not what is shown in the figure.

2. Fig. 7AC need statistical analysis (as do luciferase results in the supplement).

3. Fig. 7B – how can you get a DNA binding logo from a single sequence which is what is presumably used in the luciferase assay?

4. Fig. 3G and line 240. It is not clear if RXR binds the half sites nor whether the heterodimer is necessary for binding. Do they see binding of PPARg alone to half sites?

5. Fig. 6 ChIPseq and RNAseq analysis – These data need to be deposited in GEO along with the PBM data. It should also be deposited in Transcriptomine on the NURSA website. The analysis is a bit simplistic (based only on whether a site is within 10kb upstream of a gene that is activated by a ligand) but the result is clear. A similar analysis could be applied to PPARg binding to DR4 and LXR binding to DR1s, potentially providing genome-wide support for the luciferase results in Fig. 7 that show that on a handful of sites PPARg activates best from DR1s even if binds to DR4s, sometimes, in the PBMs. In Fig. 6CD – it is not clear if half site or full sites sequences are being examined. A distinction between those types of sites would be useful.

6. Lines 228-229 – Suppl Table S2 just gives the antibodies used, not the data

7. There are some differences between the methods used for the luciferase assays and the PBMs that should be addressed as they could affect the results and should be addressed:

a. Presumably no ligands are used in the PBMs as there is no mention of them. Binding ligand could allosterically change the NR to bind different DRs.

b. The His tag was removed for the PBM experiments but presumably not for the luciferase experiments. His-rich regions can bind DNA and could affect DNA binding and transactivation.
c. NRs produced in bacteria do not always have the same binding affinity as do when produced in mammalian cells. Whereas clearly they are getting binding to sites that make sense in the PBMs the

possibility of differences in binding specificity and affinity in bacterial v. mammalian expressed proteins needs to be acknowledged.

8. Fig. 2 – in DR2-5 there is no central sequence analogous to the CAAAG in DR1. This might be worth mentioning.

9. Fig. 3 – just because there is no effect of a SNV on a given half site, it does not mean that the protein does not make contact with the DNA. Indeed, the opposite is often the case –when a protein binds more weakly to DNA, a consensus sequence is typically much more pronounced as the bases need to be exactly correct in order to see binding. In contrast, when a protein binds well to DNA, a consensus motif may be not be obvious as the protein does "not care" what sequence it binds to – it will bind any DNA

10. Fig. 3E, l. 207-210. A z-score is for a single sequence but the logos are derived from more than 1 sequence

11. L.234 -- the 38 bound sequences does not make sense as the previous line says that 38/39 do NOT bind DNA. It would also be useful to have the 38 sequences discussed in Fig. 3G in a table somewhere with an indication of the different categories. Fig. 3G – it would be useful to add the numbers 38,15,34 to the logos to follow the text.

12. Lines 304 to 313 – this discussion is a little confusing. How do you generate a binding logo for EACH seed sequence? Do they mean for each SET of seed sequences? How do we know that a full or a half-site binding mode is being contacted for a given DR sequence?

13. Fig. 5 –Need to indicate exactly what is being plotted in the scatter plots: Average PBM binding score from replicates or from groups of sequences related to a given seed sequence? Presumably it is from the latter, as you cannot get a logo from a single sequence. Line 322-323 – not clear what is meant here. Line 339-341 – are DR1 and DR4 distinguished in 5F?

14. Line 339-341 – where is this shown?

15. Lines 441-443 – there is another PBM paper on NRs that showed in 2012 that NR binding specificity is not determined solely by the spacer and that there can be a great deal of variability in NR binding sites: PMID: 22383578. It should be cited.

16. Discussion – consider redefining NRs as group II heterodimeric partners with RXR –statements such as those on lines 463-465, if read on their own, could be misleading.
17. The Discussion is rather long and repetitive in parts. It could be shortened.

Minor formatting issues
1. Fig. 1 – In addition to the probe design, the 24 seed sequences need to be indicated someplace. Are the SNVs in the half sites as well as the flanking region? A schematic or summary of the PBM design in the supplement would be useful. (I could not access the file with all the PBM data so perhaps it is there)

2. Fig. 2 – is the line in the middle of the logo needed?

3. L.219: are should be may

4. L. 307-311 – need to reference Fig. 5A

5. Supplemental Table S1 – column description and legends are cut off

6. Supplemental Table S2 ideally would have references for the sequences from the literature
7. Figure legends do not have titles

Reviewer #3 (Remarks to the Author):

This manuscript authored by Penvose et.al. tackles and important challenge in transcription factor biology, which is to relate DNA binding affinity to in-cell activity. This is important since for some NRs the preferred DNA binding motifs identified in literature explain <10% of ChIPseq data. This study focuses on type II nuclear receptor-DNA interactions and how affinity and selectivity relate to transactivation. High-throughput competitive protein binding arrays (PBMs) data is compared to Chip-seq datasets to link binding mode and preference with gene expression. Results are validated in a strait forward way with simple LUC assays. The impact of this work includes showing that TypeII NRs are able to bind as homodimers to half-sites, TypeII NRs bind to elements with a range of half-site spacings, in vitro specificity better correlates to DNA binding site activity than binding affinity. All experiments used full-length NRs which also increase relevance. While it is known that NRs can bind half-sites, showing that all NR heteromdimers tested bind half-sites and that both RXR and its partner can perform this function is interesting. The findings of this manuscript revise and update the current model of NR binding to DNA and further suggests more promiscuous binding for NR with different DR spacer length. Overall, this is a well-designed study and the authors provide sufficient results to support their claims. These reductionist approaches are critical to link biochemical/ biophysical behavior (in vitro) to function in cells helping the field to determine what translates from the test tube to biology.

Figures are self-descriptive and nicely labelled. Although, I felt that the manuscript be shortened. There is some redundancy in the results section with the discussion section. A tight and concise description of results could enhance impact. The discussion section covers the most significant conclusions of the study in context of current available literature in the field.

Is there a correlation between full NR dimeric binding sites and half sites in the direction of gene expression? Could this be determined for data sets with ChIPseq and corresponding RNAseq data?

PPARy on line 459 should be PPARgamma.

We thank the referees for their time and thoughtful suggestions. We have addressed all questions and concerns raised by referees below on a point-by-point basis.

Reviewers' comments:

Reviewer #1 (Remarks to the Author):

Reviewer: Penvose, et al. examine the sequence specificity of Type II nuclear receptors (NR) that bind DNA as heterodimers with a common partner (RXRa). While confirming the preferences for known direct repeat(DR) binding site arrangements, they also report unexpected promiscuity in binding of all 11 heterodimers and the RXRa homodimer to the wider set of DRs (repeats spaced by 0-5 intervening bases). By motif building, site ablation and protein mutation they demonstrate that heterodimers likely bind to different DR arrangements (DR 0-5) by associating with a single repeat or a half site. The two modes of interaction neatly explain the genome-wide ChIP-seq studies which often find that a small fraction of the ChIP peaks contain an ideal DR repeat and much larger fraction contains half sites. The authors go on examine the role of half site binding in ligand-responsive reporter gene expression. Their results confirm that half site binding may permit inducible gene expression but optimal DR sites may result in higher ligand-responsive expression of the reporter. Their data lead them to draw a distinction between high affinity sites and high activity sites, emphasizing that high affinity in vitro or in vivo does not predict higher gene-induction in cells.

Taken together this study provides an important step forward in understanding the function of type II NRs in cells, therefore it certainly merits publication. However, some of the results do raise concerns that should be addressed to ensure that interpretations are made on data that are not influenced by the experimental platform or computational models that were utilized in this study.

An important technical concern is that promiscuity in binding to different DR sites may be a consequence of performing the binding experiment on the protein-binding microarray (PBM) platform. For example, the density of DNA probes on the surface and low rate of mass transport at surfaces generate non-equilibrium conditions and may enhance NR binding to sites that may not otherwise be bound in solution. Moreover, low level aggregation of recombinant NRs (given the 3:1 ratio of the two partners) may enable multi-site binding across different DNA probes reducing the discrimination between different DRs. To address these type of platform-based concerns, it would be necessary to demonstrate binding to the range of binding sites using other methods such as electrophoretic mobility shift assays or solution-based fluorescence polarization assays. Different concentrations of the protein complex should be tested to determine if their PBM-

based DR promiscuity diminishes at lower protein concentrations or more stringent in vitro binding conditions.

Authors Response: We agree strongly with the reviewer that our results would be even stronger if validated by an independent method, to ensure that the binding promiscuity observed is not due to a technical artifact of the PBM. To address this we have performed competition EMSAs for PPARg:RXRa heterodimer to different DNA sites over a wide affinity range. This data is now included in Figure 1 and Supplementary Figure 1. We observed excellent agreement for the relative binding preferences determined by both PBM and EMSA experiments, demonstrating that our observed promiscuity is not an artifact of the PBM, and that PBMs accurately reflect binding observed in solution (i.e. by an orthogonal method).

Critically, to specifically address the reviewers concern and validate the promiscuous half-site binding found by our PBM analysis, the EMSA data corroborated our observation that sequences bound in a half-site mode are affected differentially by mutations in either half-site. For example, the DR1 site P3 (Figure 1g) was shown to be bound in a half-site mode by our PBM (z-score 8.5) and was differentially affected by the 5'-half-site ablation (P3 5'-Abl z-score 0.9) and 3'-half-site ablation (P3 3'-Abl z-score 7.8). This was beautifully corroborated by our EMSA experiments (P3 k_d = 320 nM; P3 5'-Abl k_d = 17,341 nM; P3 3'-Abl kd=953 nM). These experiments now demonstrate that the alternate binding modes observed in our PBM experiment are validated by an orthogonal methodology.

To address reviewers concerns about potential promiscuous binding (i.e., the wide-spread half-site motifs) due to high sample concentration or orientation of the binding site in the PBM probe we have now included (1) the corroborating experiments with EMSA that show agreement with our PBM data (see above), (2) have performed PPARγ:RXRα PBMs at decreasing concentrations (new Supplementary Figure 3), demonstrating that we observe the same full and half-site motifs at a wide range of concentrations, well below those used in standard PBMs, and (3) demonstrated agreement between motifs derived from binding sites in either orientation on the PBM. This is also now discussed in the main text on lines 190-205, "To ensure that the widespread half-site binding identified by our PBM experiments were not a result of our methodology we performed several analyses. First, we tested whether half-site binding was due to the orientation of the NR binding site within the PBM probe with respect to the microarray slide. We found that both full and half-site modes were identical for binding sites in either orientation on the PBM (**Supplementary Fig. 2**). Second, we tested whether half-site binding was affected by the protein concentration by performing PBMs at successively lower concentrations and found identical DNA binding logos at all concentrations (**Supplementary Fig. 3**). Finally, we used EMSA experiments to test the differential impact of base mutations on a DNA site

bound in half-site mode (**Fig. 1g,** sequences P3, P3 5'-Abl, P3 3'-Abl). Critically, the binding mode of PPARγ:RXRα determined by our PBM experiments (i.e., a 5' half-site mode) was corroborated by EMSA experiments (i.e., 5' half-site ablation greatly reduced binding whereas 3' half-site ablation only modestly affected binding) (**Fig. 1g and Supplementary Fig. 1**). These results demonstrate that PBM-derived binding modes accurately represent native NR binding modes."

Reviewer: While the authors examine binding of each partner to the set of PBM probes and report high correlation, suggestive of heterodimer binding, it is important to demonstrate that heterodimers are forming and to isolate heterodimers by standard chromatographic processes and validate the binding profiles for at least a handful of proteins that have been reported to binding DNA as monomers. This is particularly important because of the high level of half site binding that they observe. Both NRs and RXRa can bind similar half sites with similar affinities.

Authors Response: We agree fully with the reviewer's concerns about ensuring heterodimer binding. We rationalized that demonstrating the heterodimer formation on an independent platform (i.e., chromatography) would not specifically demonstrate that the heterodimers were binding in the PBM experiment, as the biophysical context is considerably different and may not translate. We outline the steps taken in our original and revised manuscript to demonstrate that we are observing heterodimer binding in the PBM and to address the reviewer's concerns. Most critically, we ensured that the PBM results for all heterodimers were identical when protein binding was probed with antibody to either the RXRa or the other NR proteins. In other words, to assay binding of the PPARg:RXRa heterodimer, we required that the results were consistent when using an anti-PPARg and an anti-RXRa antibody. We have altered the main text to more directly describe this critical control experiment, and have included the homodimer PBM control data (Supplementary Figure 5). The text on lines 123-130 now reads, "To ensure heterodimer binding we required that the binding results agree when performed using antibodies to RXRα or the non-RXRα partner. Binding profiles using separate antibodies showed strong correlation, demonstrating that both protein partners were bound to each DNA probe at identical levels (**Fig. 1d,** R^2 of antibody replicates in **Supplementary Table 1**). Binding of homodimers were not correlated with each other, nor with the heterodimers (**Supplementary Fig. 5**), further demonstrating heterodimer binding."

Most NR homodimers do not bind DNA with high affinity; however, the THR homodimer can bind DNA well. We initially found that the 3:1 ratio led to appreciable homodimer binding in our heterodimer experiment, which was seen as a poor correlation between the different antibodies (ie., THR and RXR Abs). To remedy this we reduced the ratio to 1:1 and found much better agreement. In the revised manuscript we now provide comparisons of the homodimer binding

for several NRs (THR and VDR, which are known to function as homodimers, are included) as Supplementary Figure 5, and provide all homodimer binding as Supplementary File 1 (and in NCBI GEO GSE 124910).

Finally, we believe that the strong agreement between the PBM data and the reviewer suggested EMSA experiments (see above) also now addresses this technical concern. We observed only a single shifted band in our EMSA experiments (suggesting a single species) and the binding agreed well with our heterodimer PBM data, demonstrating that our data was due to heterodimer binding.

Reviewer: The most striking conclusion of their study is that nearly all NRs bind half sites and this leads to high affinity associations with different DRs. To emphasize their point, the authors display one subunit of the dimer not making contact with DNA. This may be misleading as the very first crystal structure of GR homodimer by Paul Sigler revealed one monomer making base-specific contacts while the other monomer docked on to DNA without making such contacts but retaining electrostatic interactions with the DNA backbone. More important, it is unclear why 5' half sites are different than 3' half sites. Given that the sites are direct repeats and based on their probe design, the dimer should be able slide/hop over to the next repeat (5' or 3') and associate indistinguishably with the duplex. The exception to this possibility is the 5' half site preference displayed by PPARg. The authors do not adequately address this worrying anomaly. This result raises the concern that arrangement of the binding sites with respect to the glass side may influence binding to the more solvent-accessible 5' half site. The extent to which the PBM platform influences binding of NR heterodimers has not been rigorously evaluated. Previous reports (Weirauch et al Nature Biotech 2014) had reported that proximity to the slide had an impact on accessibility and could skew binding preferences. It is imperative that such half site preferences are examined by alternative methods, such as MITOMI, that eliminate any potential for biased protein associations on surface-immobilized short DNA duplexes.

Authors Response: First, we agree that the representation of the heterodimer member as not touching the DNA in our schematic may be confusing. We had initially done this only to emphasize that the orientation is different, and agree with the reviewer that the protein adjacent to the non-specifically-bound half-sites is likely making electrostatic contacts with the backbone. We have adjusted our representations in the manuscript (Fig. 3 and Fig. 7).

Second, the confusion regarding the half-site preferences is likely due to our not qualifying the term direct repeat. The half-site sequences in most DR sequences probes on the PBM are not identical. The term direct repeat (DR) follows the

more general designation in the literature in which two recognizable half-sites are oriented in a directly repeating fashion, but need not be composed of identical sequences. For most of the DR sites recognized in the half-site mode, the other half-site was a different sequence. In general, we found that there was a tendency to bind the higher-affinity half-site of the two available, but this rule was not strictly followed and may be based on the preferences of each dimer member for certain DNA sequences flanking the half-sites. To address this confusion about DR sequences we have more clearly pointed out in the main text on lines 102-105 that the half-sites are not required to be identical, "Seed sequences were generated by combining different half-site sequences exhibiting a range of degeneracy from the consensus 5'-RGKTCA-3'. Therefore, most seed sequences contain distinct half-site sequences in the 5' and 3' half-site positions."

Regarding the concern about the impact of binding site proximity to the glass slides, see above analysis of the probe orientation effects and the corroboration of our data using the EMSA experiments.

Reviewer: In an elegant series of experiments in Figure 3, the authors try to parse out which partner enables half site binding. The data in Figure 3C is clear but 3D seems redundant and best moved to supplemental materials. (On a minor note, the supplementary figures and data are poorly described and seem to have suffered some form or formatting glitch. It would be helpful to the audience to have this material more accessible.)

Authors Response: We believe that the figure in 3d supports the generality of our conclusion that half-site selectivity occurs on many different sequences bound in a half-site mode, and is not limited to a single selectively chosen sequence as is depicted in fig 3b&c. However, for brevity (following the reviewers suggestion) we have moved this panel to a supplementary figure (Supplementary Figure 4).

We have also added and expanded captions for all previous and new supplementary figures as suggested.

Reviewer: The RXRa mutation demonstrates half site binding by PPARg but it also increases the preference for TGT upstream of the half site (Fig 3G). This preference is different than in Fig 3E. Why would such a preference for 5' flanks appear when RXRa is mutated? The reciprocal mutations in PPARg and LXRa would help clarify if this is a latent preference of those particular NRs or if this is a general feature of NR heterodimers. I would highly recommend this experiment as it would also address why nearly 50% of the sites that are bound in the half-site mode permit binding by PPARg whereas the other half are only bound by RXRa. Such clear difference in the ability each partner to associate with specific half sites is extremely odd and needs to be addressed by the authors.

Authors Response: We agree with the reviewer and have performed the reciprocal experiment with a mutant PPARg protein, now allowing us to examine the impact of mutation to either dimer. We note that we were not able to generate a mutant LXRa protein that dimerized appropriately with RXRa. These new results with a mutant PPARg protein are now described in the main text (Lines 222-281) and provide additional light on the contribution from heterodimer partners to the full and half-site binding modes. Our reciprocal mutants did not specifically clarify the observation that RXR mutations lead to an increase in preference for the TGT upstream, as the PPAR mutation leads to a more severe reduction in binding and abrogates most of the half-site binding. We believe the increase in the TGT base preference may be a compensation for a loss in non-specific DNA interactions from RXR, but are unable to definitively explain this change. We thank the reviewer for this suggested experiment as we believe our expanded analysis now strengthens our paper.

Reviewer: If differences between half sites are not apparent from their own computational method, the authors may consider methods such as No-reads left behind (Bussemaker) to see if other NR-specific specificity determinants are identified that explain the differential association of each partner with different half sites. NRLB may eliminate the need for an arbitrary z-score cut-off (>3) and reveal specificity determinants that might be more evident in lower affinity sites.

Authors Response: We thank the reviewer for this suggestion. We looked into the No-reads-left-behind (NRLB) algorithm, and found that it will not work on our dataset as the number of sequences is too limited for such a 'learning' algorithm as the data is not 'count based' as one gets from sequencing-based approaches (personal communication R. Chaitanya, developer of NRLB algorithm). Similar concerns existed for other available algorithms.

We note that our approach was not to learn motifs computationally using a large diverse dataset, as we were unable to include enough sequences at all DR spacer lengths on a single PBM to do so. In preliminary experiments we tested the ability of support-vector machines (SVMs) to learn NR binding motifs and found that the required number of sequences made this approach prohibitive for our task of a comprehensive survey. Furthermore, we found that the approach of directly measuring and representing the impact of SNVs on binding (the one used in the manuscript) lead to more robust motifs.

Reviewer: Given the recent emphasis on the importance of DNA shape, it is unclear why the authors did not explore the potential role for shape in explaining the different preference of VDR for different DR3 sites or LXRa for different DR4 sites. It would enrich their study to include this analysis of the spacer. Similar

analysis of Hox-Exd heterodimers by the Bussemaker and Mann labs revealed latent preferences of different Hox factors.

Authors Response: We agree with the reviewer that DNA shape is an important aspect of TF-DNA binding. We have now included an analysis of DNA shape parameters that might contribute to VDR and LXRa binding preference for DR3 and DR4 sites as suggested. Lines 375-394 in the main text now read, "To examine the contribution of the spacer sequence to NR specificity we examined how spacer variants can modulate NR-DNA binding (**Fig. 5g**). We focused our analysis on NRs that exhibit preferences for DR3 and DR4 sites. Examining the binding affinity distribution for SNVs within the spacer of a single seed sequence, we found that the spacer sequence can have considerable impact on binding affinity in a NR-specific manner (**Fig. 5g**). NRs are known to make DNA contacts with the spacer sequence; for example, LXRβ makes minor groove contacts with DNA in the LXRβ:RXRα-DNA X-ray crystal structure [36]. Given the established role for DNA shape in TF binding specificity [37-39], we investigated whether DNA shape features in the spacer sequence might also contribute to the selectivity for different binding sites. We examined DNA shape features for spacer variants of DR3 and DR4 sites that enhance or diminish the binding of LXRα and VDR (**Supplementary Fig. 7**). The examined DNA shape features (i.e., major groove width, helix twist, propeller twist, and roll) were nearly identical for all comparisons. However, we observed a significant difference in the major groove width and roll parameters for VDR binding to DR3 sites. Our results suggest that DNA shape may also play a role in NR binding specificity. Future studies that more exhaustively sample spacer sequences may enable still more subtle differences to be identified."

Reviewer: Finally, there are a few minor issues that should be addressed. These include, reporting excessive p-values as in Fig. 6A, overstating the differences between z-scores as in the case of Fig. 7C, and arbitrary definition of "functional sites" (searching for motifs 10Kb upstream of start sites and ignoring intronic enhancers or motifs that lie beyond 10kb).

Authors Response: The p-values reported for figure 6A were directly calculated as described. We have interpreted the Reviewer's concern to be related to multiple hypothesis testing. We have now moved the p-value to the Figure legend and adjusted it accordingly using the Bonferroni correction. If we have misunderstood this point we would be happy to further modify as needed.

We have altered the wording in our description of relative z-scores in Figure 7C so as not to overstate the point. We have changed, "high-affinity DR1 sites for PPARy (DR1.8, DR1.3) show less activity than the three DR4 sites all bound with lower affinity" to "Second, for PPARγ, several high-affinity DR1 sites (DR1.8, DR1.3) show comparable or less activity than the three DR4 sites all bound with

comparable or lower affinity" on lines 464-466.

We have addressed the definition of 'functional sites' in our genomic analyses by also performing the analyses with alternate genomic windows (i.e., including 10kB up and downstream of TSS, and 50kB upstream) and find consistent results, supporting our findings. These additional analyses are now included as Supplementary Figure 8 and are described in the main text on lines 426-428, "These same trends were observed when we used alternate genomic constraints to define functional sites (i.e., 10 kbp up- and downstream, or 50 kbp upstream) (**Supplementary Fig. 8**)."

Reviewer #2 (Remarks to the Author):

Reviewer: The authors use the powerful protein binding microarray (PBM) technology to examine the DNA binding specificity of 12 nuclear receptors that bind DNA as heterodimeric partners with RXR. More than 25 years ago the DR (direct repeat) rule for NR DNA binding was formulated based on a handful of DNA binding sites that had been identified for various NRs at the time. It was an incredibly insightful breakthrough that allowed the field to deal with the complexity of 100s of NRs found in every animal organism and their 10,000s of target genes. However, this elegant rule did not address the biological reality that several NRs bound the same type of DR motifs (e.g., DR1, DR2, DR3 etc) and yet had very different functions in vivo, nor the fact that calling a given site a DR1,2,3, etc versus a variation on a single DR type motif was often in the mind of the beholder. Furthermore, nearly all the classical in vitro studies were dependent on gel shift assays; polyacrylamide gels and substantial electrical fields are not found in the nucleus. The advent of in vivo genome-wide DNA binding methods such as ChIPseq did not necessarily clarify things as predicting exactly which DNA sequence of 12-15 nucleotides is responsible for the binding of a NR to region of DNA 100 or more nucleotides is not an exact science, even though there are plenty of algorithms that predict binding motifs.

In this work the authors correlate in vitro PBM DNA binding data to 10,000s of sequences with luciferase assays as well as in vivo ChIPseq and RNAseq data to dig deeper into the DR rule for NRs. Whereas they find compelling evidence for the DR rules, they also find exceptions as well as binding to what appears to be half sites, although a cursory analysis suggests that the canonical sites based on the DR rules are the most active in vivo. Overall the manuscript is very well written and easy to follow; however, there are several major as well as minor issues that need to be addressed.

Major issues

1. The conclusions from Fig. 7 and the presentation of the model in Fig. 7D are not consistent with their data, and the text is often confusing. The luciferase data in Fig. 7AC show that even though certain half sites yield good binding in vitro in

the PBMs they do not result in good activation of transcription in vivo in luciferase assays. This is consistent with the data presented in Fig. 6CD (although it is not clear that half versus full-sites have been distinguished in these data). In contrast, in the model in Fig. 7D, they show the same level of activation by half sites as by full sites. There are also several contradictory statements in lines 396 thru 402 and lines 449-450 do not make sense. Lines 415-416 – this is not what is shown in the figure.

Authors Response: We thank the reviewer for identifying this potential confusion. We have adjusted the schematic depiction in Figure 7D to indicate functionality (i.e., with a check mark), but without indicating quantity (i.e., number of squiggles). To address any potential confusion with the text, we have added clarifying statements in the main text when we discuss the Figure 7 data, and in the discussion where we discuss our model Figure 7d on lines 486-490.

We have attempted to clarify any contradictory statements in lines 396-402 "We found that LXRa can strongly induce gene expression, in a ligand-specific manner, from a DR1 site (DR1.7) that is bound in a half-site mode (Figure 7a-b). Ablating the 5' half-site sequence (DR1.18) abrogated binding and drastically reduced reporter gene expression. As expected, ablating the 3' half-site did not affect binding affinity; however, it strongly affected reporter gene expression". We have re-phrased the text on lines 448-450 as follows, "Ablating the 3' half-site (DR1.17) did not affect binding affinity; however, unexpectedly, it strongly affected reporter gene expression, demonstrating that *in vitro* affinity does not necessarily predict binding site activity."

We have attempted to clarify the confusion in lines 449-450 that read, "We find that the canonical NR half-site preferences better reflect activity rather than DNA-binding sites affinity." We have changed the text on lines 483-486 to more appropriately indicate that the canonical preferences are for different *spacer* lengths, "We demonstrate that NR binding site activity does not follow binding affinity, and that the canonical NR DR spacer-length preferences (e.g., preference of PPARγ for DR1 sites) better reflect activity rather than DNA-binding site affinity"

We have addressed the issue indicated for lines 415-416 that read "First, LXRa can promote expression from the DR1.7 sites (Figure 7A) more effectively than from the three DR4 sites (Figure 7C), but with less pronounced agonist-dependent inducible expression." We have changed the text on lines 460-464 as follows, "First, LXRα can promote expression from the DR1.7 site (**Fig. 7a**, 40-fold activation in agonist-treated condition) at a comparable or higher level than the three DR4 sites (**Fig. 7c**, 8- to 28-fold activation in agonist-treated conditions; differences with p-values ~$1x10^{-6}$ and $1x10^{-3}$, respectively)."

Regarding whether the half- versus full-sites are distinguished in Figure 6CD, the

motifs used in the genomic searchers are the full DR1 sites and not the half-sites as described in Panels A and B. However, we now include the full data with all DR and half-site motifs as Supplementary File 8.

Reviewer: 2. Fig. 7AC need statistical analysis (as do luciferase results in the supplement).

We have now included p-values in the Figure and main text for those significant reporter gene differences that we explicitly discuss in the manuscript.

Reviewer: 3. Fig. 7B – how can you get a DNA binding logo from a single sequence which is what is presumably used in the luciferase assay?

Authors Response: We thank the reviewer for highlighting what is a potential confusion with our method for logo generation and representation. We have used an approach to generate logos based on an initial seed sequence and all associated single-nucleotide variants (SNVs) of this seed. This approach essentially assays and visually represents the impact of base changes on binding to that particular seed sequence. We therefore refer to this as a 'logo for that seed sequence' even though it was generated by binding data for the seed sequence *and all SNV sequences.* We can also generate a single logo for a factor by averaging over these single-seed logos, as was done for Figure 2.

To better explain this concept, we have expanded Figure 1c to schematize how we generate logos for single seeds using our SNV probes. We have also more specifically indicated this now at several places in the main text.

Results section, lines 105-111: "To assay NR binding specificity for each seed sequence we also measured binding to *all possible* single-nucleotide variants (SNVs), with each SNV included as a separate probe on the PBM (**Fig. 1c**). This SNV-based approach allows us to generate a binding logo (i.e., energy matrix or PWM) for each *individual seed* sequence by measuring the changes to NR binding caused by the perturbation of each base position (**Fig. 1c, Methods**)."

Methods section, lines 684-691: "Position frequency matrices (PFMs) and DNA-binding logos were generated for each seed sequence with z-score > 3.0 using the previously described SNV-based approach [21], using β = 15/maximum z-score. Briefly, logos for single seed sequences are generated using the binding data to each seed sequence and all the single-nucleotide variant (SNV) sequences for that seed sequence. For a binding site of length L there will be 3xL SNV sequences. Logos for a NR binding to a specific DR spacer length are determined by averaging over the individual seed sequence logos.

Reviewer: 4. Fig. 3G and line 240. It is not clear if RXR binds the half sites nor whether the heterodimer is necessary for binding. Do they see binding of PPARg alone to half sites?

Authors Response: For all heterodimer experiments we ensure that binding is mediated by heterodimers by requiring that the same binding patterns are seen with antibodies to each monomer. We indicated this is the main text on lines 228-230, but have added additional text to further clarify. Lines 226-230 now read, "Binding of PPARγ:RXRα-DBDmut was highly correlated using either anti-RXRα or anti-PPARγ antibodies (R^2 of antibody replicates given in **Supplementary Table 1**) showing that the RXRα-DBDmut protein formed a heterodimer with PPARγ, and that all DNA sites were bound as a mutant heterodimer." Aditionally, we have included all the homodimer binding data in Supplementary File 1. This data demonstrates that the homodimer binding landscape differs significantly from the heterodimer binding landscape (Supplementary Fig. 5).

Reviewer: 5. Fig. 6 ChIPseq and RNAseq analysis – These data need to be deposited in GEO along with the PBM data. It should also be deposited in Transcriptomine on the NURSA website.

Authors Response: The ChIP-seq and RNA-seq data are previously published datasets from other labs. We have more explicitly highlighted this is the main text and methods sections as follows. Lines 400-403 in the main text read, "Examining published PPARγ binding data in HT29 colorectal cancer cells (GSE77039) [15], we found that all PPARγ models (DRs and half-sites (HS)) could discriminate bound regions from unbound."

Lines 803-807 in the Methods now read, "To examine motif enrichment for putative 'active' sites near differentially expressed genes, RNA-seq data from HT29 cells [15] were used to identify regions that are likely to be actively controlling transcription. We re-analyzed the published RNA-seq data using DESeq2 [57] to identify genes upregulated upon agonist treatment compared to vehicle only (DMSO)."

Reviewer: The analysis is a bit simplistic (based only on whether a site is within 10kb upstream of a gene that is activated by a ligand) but the result is clear. A similar analysis could be applied to PPARg binding to DR4 and LXR binding to DR1s, potentially providing genome-wide support for the luciferase results in Fig. 7 that show that on a handful of sites PPARg activates best from DR1s even if binds to DR4s, sometimes, in the PBMs.

Authors Response: We have re-performed our genomic analysis using alternate criteria to define functional sites (i.e., 10 kbp up- and downstream, and 50 kbp upstream, new Supplementary Figure 7) and the results remain the same. This is now explicitly noted in the main text on lines 426-428, "These same

trends were observed when we used alternate genomic constraints to define functional sites (i.e., 10 kbp up- and downstream, or 50 kbp upstream) (**Supplementary Fig. 8**)."

In our re-analysis and new Supplementary Figure 7 we have included the PPARg binding to DR4 and LXR binding to DR1 as suggested. The data further confirms our hypothesis of functional specificity. We now note this in the main text. We thank the reviewer for this helpful comment.

Reviewer: In Fig. 6CD – it is not clear if half site or full sites sequences are being examined. A distinction between those types of sites would be useful.

Authors Response: In our re-analysis (described above) we now explicitly include the data for half-site motifs and demonstrate that they are not enriched in functional sites.

Reviewer: 6. Lines 228-229 – Suppl Table S2 just gives the antibodies used, not the data

Authors Response: Lines 228-229 "PBM data for PPARg:RXRa-DBDmut were highly correlated using either anti-RXRa or anti-PPARg antibodies (**Supplementary Table 1**)". The final column of our submitted Table S1 provides the R^2 for the comparison of the two PBM experiments. We have clarified that we were referring to the R^2 and not the data provided in supplementary data 1.

Reviewer: 7. There are some differences between the methods used for the luciferase assays and the PBMs that should be addressed as they could affect the results and should be addressed:

a. Presumably no ligands are used in the PBMs as there is no mention of them. Binding ligand could allosterically change the NR to bind different DRs.

Authors Response: We thank the reviewer for identifying this oversight. We performed several PBM experiments with and without ligand, and found no effect on NR binding. Therefore, all experiments were performed without ligand. We now explicitly state this is the text on lines 654-657, "Preliminary PBM experiments for PPARγ:RXRα and RXRα were performed with and without the ligands rosiglitazone and 9-*cis* retinoic acid, respectively, and we found no change in NR binding; therefore, all experiments are performed in the absence of ligand."

Reviewer: b. The His tag was removed for the PBM experiments but presumably not for the luciferase experiments. His-rich regions can bind DNA and could affect DNA binding and transactivation.

Authors Response: We agree with the reviewer that protein tags might affect DNA binding. However, we have peformed some PPARg:RXRa heterodimer PBMs with RXRa with a cleaved His-tag (Figure 1) and with an intact His-tag (Supplementary Figure 3, new to the revised manuscript), and observed no change in the binding specificity suggesting that the His-tag does not strongly affect NR binding. For our reporter gene experiments, if the His-tag does affect transcription we believe that it will do so equally for all sites and therefore our conclusions about activity differences between sites, and the lack of uniformity between PBM data and activity, should be supported.

Reviewer: c. NRs produced in bacteria do not always have the same binding affinity as do when produced in mammalian cells. Whereas clearly they are getting binding to sites that make sense in the PBMs the possibility of differences in binding specificity and affinity in bacterial v. mammalian expressed proteins needs to be acknowledged.

Authors Response: We have acknowledged this potential issue in the first results section on lines on lines 141-144, "Our protein samples were produced in bacterial or insect cells and may differ from proteins produced in a native mammalian context; however, our ability to capture known NR binding specificity suggests our data reflects native mammalian dimer binding specificity."

Reviewer: 8. Fig. 2 – in DR2-5 there is no central sequence analogous to the CAAAG in DR1. This might be worth mentioning.

Authors Response: We have now mentioned this in the main text on lines 371-375, "In NR binding logos, we observed base preferences in the spacer sequence between DR half-sites (e.g., Fig. 2; Fig. 5f, positions 12-15). We noted a strong preference for an adenine in the spacer of DR1 sites, which has been demonstrated for other NRs [32]; however, such a distinct base preference is absent at longer spacer lengths (DR2-DR5)."

Reviewer: 9. Fig. 3 – just because there is no effect of a SNV on a given half site, it does not mean that the protein does not make contact with the DNA. Indeed, the opposite is often the case –when a protein binds more weakly to DNA, a consensus sequence is typically much more pronounced as the bases need to be exactly correct in order to see binding. In contrast, when a protein binds well to DNA, a consensus motif may be not be obvious as the protein does "not care" what sequence it binds to – it will bind any DNA.

Authors Response: We agree with the reviewer that the depiction in Figure 3 of one monomer not contacting the DNA may be misleading. We had originally represented the monomer this way to highlight an alternate conformation. We have changed the figure to avoid this interpretation and simply to highlight a

potential alternate orientation of the monomer with DNA.

Reviewer: 10. Fig. 3E, l. 207-210. A z-score is for a single sequence but the logos are derived from more than 1 sequence

Authors Response: We thank the reviewer again for highlighting this issue of logos for single sequences. As described above, we have now clarified the notion of generating logos for seed sequences, which involves binding data to the seed sequences and all SNV sequences (i.e., and not a single sequence).

Reviewer: 11. L.234 -- the 38 bound sequences does not make sense as the previous line says that 38/39 do NOT bind DNA. It would also be useful to have the 38 sequences discussed in Fig. 3G in a table somewhere with an indication of the different categories.

Authors Response: We have clarified the preceding sentence in the main text to more explicitly state that the 38 sequences are now no longer bound in a full-site mode, but may still be bound in an alternate mode. Lines 250-252 read, "As expected, nearly all seed sequences (38/39) that PPARγ:RXRα bound in a full-site mode were no longer bound in a full-site mode by PPARγ:RXRα-DBDmut." We believe that this resolves the confusion. We have also added the suggested table as Supplementary Table 4.

Reviewer: Fig. 3G – it would be useful to add the numbers 38,15,34 to the logos to follow the text.

Authors Response: These logos are generated from single representative seed sequences, and are not generated by averaged/compiling over multiple sequences. We hope that our added text regarding motif generation (see above and below) now clarifies this confusion. We have also added the single seed sequences used to generate the logos in Figure 3. The curation of the different transitions for the DBDmut experiments have also now been provided as supplementary table 4.

Reviewer: 12. Lines 304 to 313 – this discussion is a little confusing. How do you generate a binding logo for EACH seed sequence? Do they mean for each SET of seed sequences? How do we know that a full or a half-site binding mode is being contacted for a given DR sequence?

Authors Response: We apologize for this confusion. As discussed above, we have added clarifying statements to the main text regarding generating logos from single seed sequences.

Reviewer: 13. Fig. 5 –Need to indicate exactly what is being plotted in the

scatter plots: Average PBM binding score from replicates or from groups of sequences related to a given seed sequence? Presumably it is from the latter, as you cannot get a logo from a single sequence.

Authors Response: We thank the reviewer for suggesting this clarification. We have more explicitly detailed what is being plotted in the scatter plots in the corresponding figure legends for Fig1 lines 1035-1036 "Dots represent average over ~5 replicates for all 10,728 1035 unique SNV probes (black dots) and 500 background probes (grey dots)" and 5 lines 1078-1079 "Each spot is the average of ~5 replicates for each unique DNA sequence (~1600 at each spacer length) on the PBM"

Reviewer: Line 322-323 – not clear what is meant here. Line 339-341 – are DR1 and DR4 distinguished in 5F?

Authors Response: Line 322-323 previously read, "..demonstrating that NRs can display preferences for particular DR spacings but that these preferences do not require a more general difference in DNA base specificity".. We have altered the text to clarify our point on lines 346-348, "This example illustrates that NRs can bind identically on one DR spacing while exhibiting binding differences on another DR spacing." Figure 5f shows binding energy logos on a DR4 sequence as described in the figure legend

Reviewer: 14. Line 339-341 – where is this shown?

Authors Response: We thank the reviewer for identifying this oversight. We have now provided the motifs for the DR1 sites as Supplementary Figure 6, we also include the DR4 motifs shown in Fig 5f in Supplementary Figure 6 to allow for easy comparisons. The lines being referred to are now lines 364-367 and include a reference to this new supplementary figure, "We 364 note that the highly unfavorable G10 preference for DR4 sites (Δz-score = -3.21) 365 is not observed for DR1 sites (Δz-score = -0.65), demonstrating that this NR-366 specific preference is not shared across all DR sites (**Supplementary Fig. 6**)"

Reviewer: 15. Lines 441-443 – there is another PBM paper on NRs that showed in 2012 that NR binding specificity is not determined solely by the spacer and that there can be a great deal of variability in NR binding sites: PMID: 22383578. It should be cited.

Authors Response: We apologize for this oversight as we were aware of the paper mentioned and had intended to cite it. We have now added the following in the Discussion section on lines 480-483, "Our findings agree with other PBM-based studies of homodimeric NRs that demonstrated near identical binding for RXRα and COUP-TF2 [41], and found that NR specificity does not solely depend on DR spacing rules [32,41]."

Reviewer: 16. Discussion – consider redefining NRs as group II heterodimeric partners with RXR –statements such as those on lines 463-465, if read on their own, could be misleading.

Authors Response: We have added text to the discussion section to re-assert that the NRs in our study are the type II dimers with RXR. For instance, line 545-547 now read, "An unexpected finding in our analysis is that all type II NR heterodimers have the ability to bind DNA in a half-site mode to either DR sites or to half-sites."

Reviewer: 17. The Discussion is rather long and repetitive in parts. It could be shortened.

Authors Response: We have reduced the Discussion from 1,453 words to 1,125 (22% reduction), and attempted to remove any duplicate statements/discussions. We thank the reviewer for this suggestion as we believe that discussion is much better in this more concise format.

Reviewer: Minor formatting issues
1. Fig. 1 – In addition to the probe design, the 24 seed sequences need to be indicated someplace. Are the SNVs in the half sites as well as the flanking region? A schematic or summary of the PBM design in the supplement would be useful. (I could not access the file with all the PBM data so perhaps it is there)

Authors Response: All PBM data and probe sequences (with Seed and associated SNVs probes annotated) are provided as Supplementary File 1, and are deposited in NCBI GEO (GSE124910). The design of the SNV probes is now described more clearly in the methods section as follows, "For each seed sequence, SNV probes were created that had a single nucleotide variant at each position of the DR half-sites, the spacer sequence between the DR half-sites, and in the 5 bp flanks of each site. Therefore, for a single 13 bp DR1 site (i.e., 6+6+1 = 13), including 5 bp flanks on either side, there would be 69 (i.e., 23x3) unique SNV probe sequences." We have added additional information to the descriptive schematic in Figure 1c, and described this SNV modeling more explicitly in the main text, "To assay NR binding specificity for each seed sequence we also measured binding to *all possible* single-nucleotide variants (SNVs), with each SNV included as a separate probe on the PBM (**Fig. 1c**). This SNV-based approach allows us to generate a binding logo (i.e., energy matrix or PWM) for each *individual seed* sequence by measuring the changes to NR binding caused by the perturbation of each base position (**Fig. 1c, Methods**). To capture binding preferences for flank and DR spacer sequences, we included SNVs for the five nucleotides upstream and downstream of the DR and across the spacer sequence."

Reviewer: 2. Fig. 2 – is the line in the middle of the logo needed?

Authors Response: We have removed the line from the middle of the logos in Figure 2 and thank the reviewer for the suggestion as it has improved the aesthetic of this figure.

Reviewer: 3. L.219: are should be may

Authors Response: Thank you, we have fixed this typo.

Reviewer: 4. L. 307-311 – need to reference Fig. 5A

Authors Response: We thank the reviewer for noticing this omission and have added the figure reference at the end of line 324 in the revised manuscript.

Reviewer: 5. Supplemental Table S1 – column description and legends are cut off

Authors Response: We have added the column descriptions to the main Word document now for the journal to integrate. This should resolve the issue.

Reviewer: 6. Supplemental Table S2 ideally would have references for the sequences from the literature

Authors Response: Again, we apologize for the confusion, we believe that they were cutoff in the formatting. They are present and we will fix the formatting issues with the journal.

Reviewer: 7. Figure legends do not have titles

Authors Response: We have corrected this omission.

Reviewer #3 (Remarks to the Author):

Reviewer: This manuscript authored by Penvose et.al. tackles and important challenge in transcription factor biology, which is to relate DNA binding affinity to in-cell activity. This is important since for some NRs the preferred DNA binding motifs identified in literature explain <10% of ChIPseq data. This study focuses on type II nuclear receptor-DNA interactions and how affinity and selectivity relate to transactivation. High-throughput competitive protein binding arrays (PBMs) data is compared to Chip-seq datasets to link binding mode and preference with gene expression. Results are validated in a strait forward way with simple LUC assays. The impact of this work includes showing that TypeII

NRs are able to bind as homodimers to half-sites, TypeII NRs bind to elements with a range of half-site spacings, in vitro specificity better correlates to DNA binding site activity than binding affinity. All experiments used full-length NRs which also increase relevance. While it is known that
NRs can bind half-sites, showing that all NR heteromdimers tested bind half-sites and that both RXR and its partner can perform this function is interesting. The findings of this manuscript revise and update the current model of NR binding to DNA and further suggests more promiscuous binding for NR with different DR spacer length. Overall, this is a well-designed study and the authors provide sufficient results to support their claims. These reductionist approaches are critical to link biochemical/ biophysical behavior (in vitro) to function in cells helping the field to determine what translates from the test tube to biology.

Figures are self-descriptive and nicely labelled. Although, I felt that the manuscript be shortened. There is some redundancy in the results section with the discussion section. A tight and concise description of results could enhance impact. The discussion section covers the most significant conclusions of the study in context of current available literature in the field.

Authors Response: We have moved some figure panels to the supplement (Figure 3) and have attempted to make the results and discussion sections more concise; more than 2000 words have been removed in the revised manuscript.

Reviewer: Is there a correlation between full NR dimeric binding sites and half sites in the direction of gene expression? Could this be determined for data sets with ChIPseq and corresponding RNAseq data?

We think that this is an interesting point. We have attempted to address this issue in our genomic analysis and accompanying figure (Figure 6). In this section we examine first how the full and half-site sequences compare with ChIP-seq data, and then compare how the enrichment of full or half-site sequences is affected when integrating RNA-seq data. The conclusions support our reporter gene experiments.

Reviewer: PPARy on line 459 should be PPARgamma.

Authors Response: We thank the reviewer for bringing this oversight to our attention and have corrected it.

REVIEWERS' COMMENTS:

Reviewer #2 (Remarks to the Author):

The authors have done an excellent job at addressing all of my previous concerns.

Reviewer #3 (Remarks to the Author):

The authors have provided a substantially revised manuscript thoroughly addressing our comments and concerns. I feel that this paper is now suitable for publication in Nature Communications and will have an impact on the field.
